# Bioactivity-driven prediction of antibacterial synergy using machine learning models

**DOI:** 10.1371/journal.pcbi.1014849

**Published:** 2026-10-05

**Authors:** Hannie Yousefabadi, Mahya Mehrmohamadi

**Affiliations:** Department of Molecular Biotechnology, School of Biotechnology, College of Science, University of Tehran, Tehran, Iran; CSIR-IHBT: Institute of Himalayan Bioresource Technology CSIR, INDIA

## Abstract

**Motivation:**

Combination therapies have demonstrated improved efficacy across diverse clinical settings. Predicting antibacterial drug synergy remains difficult due to strain variability and the limited scale of experimentally tested combinations. Existing machine-learning approaches for synergy prediction often rely on permissive cross-validation schemes that allow drug pairs to re-appear across folds, leading to inflated performance metrics. A rigorous evaluation framework and scalable feature representation are needed for robust generalization.

**Results:**

We assembled a curated dataset of 3,160 drug–pair–strain interactions covering 97 antibacterial compounds and 10 bacterial strains. We then developed **HALO** (*Held-out Antibacterial interaction Learning from latent bioactivity Observations*), a synergy-prediction framework in which each drug pair is encoded using multi-level Chemical Checker (CC) similarity features spanning chemical, targets, networks, cellular, and clinical bioactivity domains. Using strictly nested, drug-pair heldout cross validation with fold-internal feature selection, HALO achieved consistent generalization to unseen combinations (ROC–AUC = 0.78). Performance dropped notably under increasingly stringent heldout schemes and inflated under random data splits, underscoring the importance of rigorous evaluation for this task. Despite these conservative conditions, HALO transferred to two independent datasets, achieving ROC–AUC = 0.90 and 0.71 and average precision = 0.76 and 0.94 for distinguishing synergy from antagonism. Together, these results demonstrate that multi-level bioactivity signatures provide a scalable, interpretable basis for predicting antibacterial relationships, while clarifying the realistic performance limits of current models under truly leakage-free evaluation.

## Author summary

Bacterial infections are increasingly hard to treat as resistance to antibiotics spreads faster than new drugs are developed. One strategy to fight back is combining two drugs together, since some pairs work better together than either does alone (synergy), while others can interfere with each other (antagonism). Predicting which drug pairs will be synergistic before running costly lab experiments could speed up the search for effective combinations. However, existing computational models are often evaluated in ways that let the same drug pair appear in both training and testing data, making their reported performance overly optimistic and unreliable in practice. We built a machine-learning framework called HALO that predicts antibacterial drug synergy using multiple layers of biological and chemical similarity information, while strictly preventing any drug pair from being seen during both training and evaluation. Under this stricter, more realistic testing setup, HALO still generalized well to drug pairs it had never encountered, and its predictions held up on two independent external datasets. Our results show that combining diverse bioactivity signatures with rigorous evaluation gives a more trustworthy — if more conservative — picture of how well these models can prioritize new antibacterial drug combinations for experimental testing.

## 1. Introduction

Antimicrobial resistance (AMR) continues to rise globally and now poses a major public-health threat, with resistance spreading faster than new antibiotics are being developed [1–3]. Because the clinical pipeline remains dominated by derivatives of existing classes, combination therapy has become an important strategy for enhancing efficacy and slowing the emergence of resistance [4]. Yet antibiotic interactions are highly variable: synergy can improve killing and suppress resistance, whereas antagonism can reduce efficacy or promote failure [5–7]. Measuring these interactions at scale remains challenging—synergy assays are labor-intensive, context-dependent, and often vary across strains and conditions [8]. In addition, High-throughput screenings have indicated that most combinations clustering near additivity and true synergy or antagonism occurring infrequently across species and strains [9–11]. This variability, together with limited strain coverage and noisy measurements, has constrained the development and evaluation of predictive models for antibacterial synergy. These limitations have motivated computational approaches for prioritizing combinations. While machine-learning models have shown strong performance in oncology [12,13], antibacterial applications have been hindered by sparse, heterogeneous datasets, leading to low generalizability to unseen drug pairs [14,15]. More broadly, computational and machine-learning approaches have been applied to a range of drug-relationship and drug-target prediction tasks in pharmacology, including in silico screening for anti-parasitic drug discovery [16] and prediction of drug-molecular target associations such as drug-miRNA interactions [17], underscoring the broader utility of computational prediction frameworks in this space.

Existing computational approaches can be broadly grouped into three categories. Network- and graph-based models leverage drug–target or protein–interaction networks, but are typically trained on small datasets and evaluated under random or weakly stratified cross-validation, limiting assessment of generalization to unseen drug pairs. Metabolism- and flux-based methods integrate genome-scale metabolic models with machine learning to predict interaction outcomes, offering mechanistic insight but focusing on condition-specific settings rather than pair-level extrapolation. Structure-based chemoinformatic models, such as CoSynE (trained on 153 pairs among 18 compounds) use molecular fingerprints and have shown enrichment of validated synergies in prospective tests, yet similarly rely on evaluation schemes that allow substantial overlap between training and test drug pairs [18]. Among antibacterial-specific frameworks, INDIGO combines chemogenomic profiles with Random Forests models to predict interactions in *E. coli* (trained on 105 pairwise interactions among 15 compounds, expanded to 171 pairs with test-set inclusion), achieving moderate accuracy and partial transferability across species via orthology mapping [19]. A graph-learning model, trained on 91 pairwise combinations among 14 antibiotics, was able to outperform INDIGO and CoSynE with precision ≈ 0.875 and accuracy of 0.90 [20]. Another machine learning framework extended prediction to two and three drug combination predictions and achieved accuracy ≈ 0.82 on evaluation set [21]. However, across these methods, evaluation is generally performed under random or partially stratified cross-validation within a single strain, where many drug pairs—or close analogs—appear in both training and test sets. As a result, reported accuracies primarily reflect interpolation rather than true generalization to unseen combinations.

The **Chemical Checker** (CC) is a comprehensive drug dataset that offers a richer alternative to structure-only features by integrating chemical, biological, and phenotypic drug signatures into standardized embedding spaces [22]. Models that incorporate these bioactivity features consistently outperform purely structural descriptors [23]. CC-based frameworks—including CCSynergy, Bio-Mol, and recent repurposing studies—demonstrate that multi-level bioactivity embeddings can capture complex drug behavior relevant to combination modeling [24–27]. Existing antibacterial models integrate chemical and biological information [28], but rarely incorporate strain-level variation despite strong evidence that interaction outcomes differ across species, physiology, and strains [8,15]. As a result, generalization to unseen drug pairs or unseen strains remains largely untested. INDIGO and CoSynE predict continuous or 3-class interaction outcomes from chemogenomic or structural-fingerprint features respectively, evaluated on small single-strain datasets (91–171 pairs among 14–19 compounds); Lv et al.‘s network-based approach evaluates target-network topology rather than training a predictive classifier in the same sense. (Table 1).

**Table 1:** Comparison of HALO with prior antibacterial-specific synergy prediction frameworks.

Method	Model / architecture	Feature type	Output	Dataset scale	Strain	Evaluation scheme	Reported metrics
CoSynE [16]	Random Forest	Substructure fingerprints	3-class (syn/add/antag)	153 pairs, 18 cmpds, 1 strain	*E. coli MG1655*	K-fold / leave-drug-out / leave-pair-out	Acc = 0.81, AU-ROC = 0.77 (leave-pair-out)
INDIGO [17]	Random forest	Chemogenomic profiles	Continuous Loewe α	105–171 pairs, 15–19 cmpds (in train–test), 1 strain	*E. coli MG1655*	Leave-drug-out / leave-pair-out	*R* = 0.52 (*P* = 10 ^− 6^) for 66-pair test
Lv et al. [18]	Graph regularization + network propagation	Protein‑protein interaction network + drug targets	Binary	91 pairs, 14 cmpds, 1 strain	*E. coli MG1655*	Not confirmed	Acc = 0.90, Precision ≈ 0.875
HALO	LightGBM	Multi-level CC bioactivity embeddings	Binary	2,449 interactions, 97 cmpds, 10 strains	—	Nested pair-held-out CV	ROC-AUC = 0.78, Accuracy = 0.70 (Sec. 2.2)

To date, CC-style embeddings have not been systematically applied to antibacterial interactions, and existing antibacterial-specific frameworks differ in both feature representation and evaluation rigor. Critically, although CC-based embeddings have been explored in other therapeutic areas such as oncology and general bioactivity profiling, they have not previously been used for dedicated antibacterial synergy prediction. Existing antibacterial models use chemogenomic profiles, structural fingerprints, or target-network topology; none leverages the multi-level bioactivity representation provided by the Chemical Checker. Here we develop HALO (*Held-out Antibacterial interaction Learning from latent bioactivity Observations)*, therefore, to fill a distinct representational and methodological gap, in addition to establishing a more rigorous evaluation standard for similar tasks in general. HALO’s 2,449 labeled interactions across 97 compounds and 10 strains represent the largest and most strain-diverse dataset among directly comparable antibacterial synergy prediction frameworks, enabling the evaluation rigor summarized in Table 1.

Feature representation, prediction target, dataset scale, and evaluation design differ substantially across methods, limiting the feasibility of a strict same-dataset, same-protocol benchmark (see Discussion). Reported metrics are each method’s own published headline result and are not directly comparable across rows due to differing prediction tasks (continuous score, 3-class, or binary) and evaluation schemes.

## 2. Results

### 2.1 Data curation

We integrated interaction measurements from Brochado et al. [9], Cacace et al. [10], as well as manually curated literature-reported pairs from the Antibiotic Combination DataBase (ACDB) [29] (Fig 1A). To quantify drug interactions, these datasets use ε by integrating three experimental measurements. Under the Bliss independent model, interaction is quantified as the deviation (ε) between the observed combined fitness and the expected fitness given by the product of single-drug fitness. After standardizing drug and strain identities and restricting the compound space to approved antibacterials, Bliss ε values were harmonized across studies and consolidated using a reproducible procedure for resolving replicate inconsistencies. This yielded 3,160 high-quality drug pair–strain interactions. 711 of 3,160 pair-strain instances (22.5%; Fig 1B) fell within the neutral/additive band (−0.1 ≤ ε ≤ +0.1) and were excluded. This is consistent with established guidance in the synergy-scoring literature: widely used tools such as SynergyFinder explicitly caution that near-zero interaction scores provide limited confidence for calling an interaction synergistic or antagonistic, and adopt an analogous neutral band around additivity for this reason [30]. This is also consistent with prior antibiotic-interaction studies that treat near-zero interaction scores as a distinct, lower-confidence category rather than forcing them into a binary call [31]. This practice is also standard in synergy-scoring tools more broadly, which flag near-additive scores as providing limited confidence for either synergy or antagonism calls. We additionally note that antagonism and synergy represent the clinically actionable extremes of drug interaction — antagonism as a failure mode to avoid, synergy as an opportunity to exploit — while additivity is both the most common and least actionable outcome in large-scale antibiotic interaction screens [32], making the binary framing a deliberate alignment with clinical relevance rather than a convenience filter. Robustness of HALO’s performance to this threshold choice (sensitivity analysis to Bliss neutrality cutoff) is shown in S1 Fig.

**Fig 1:**
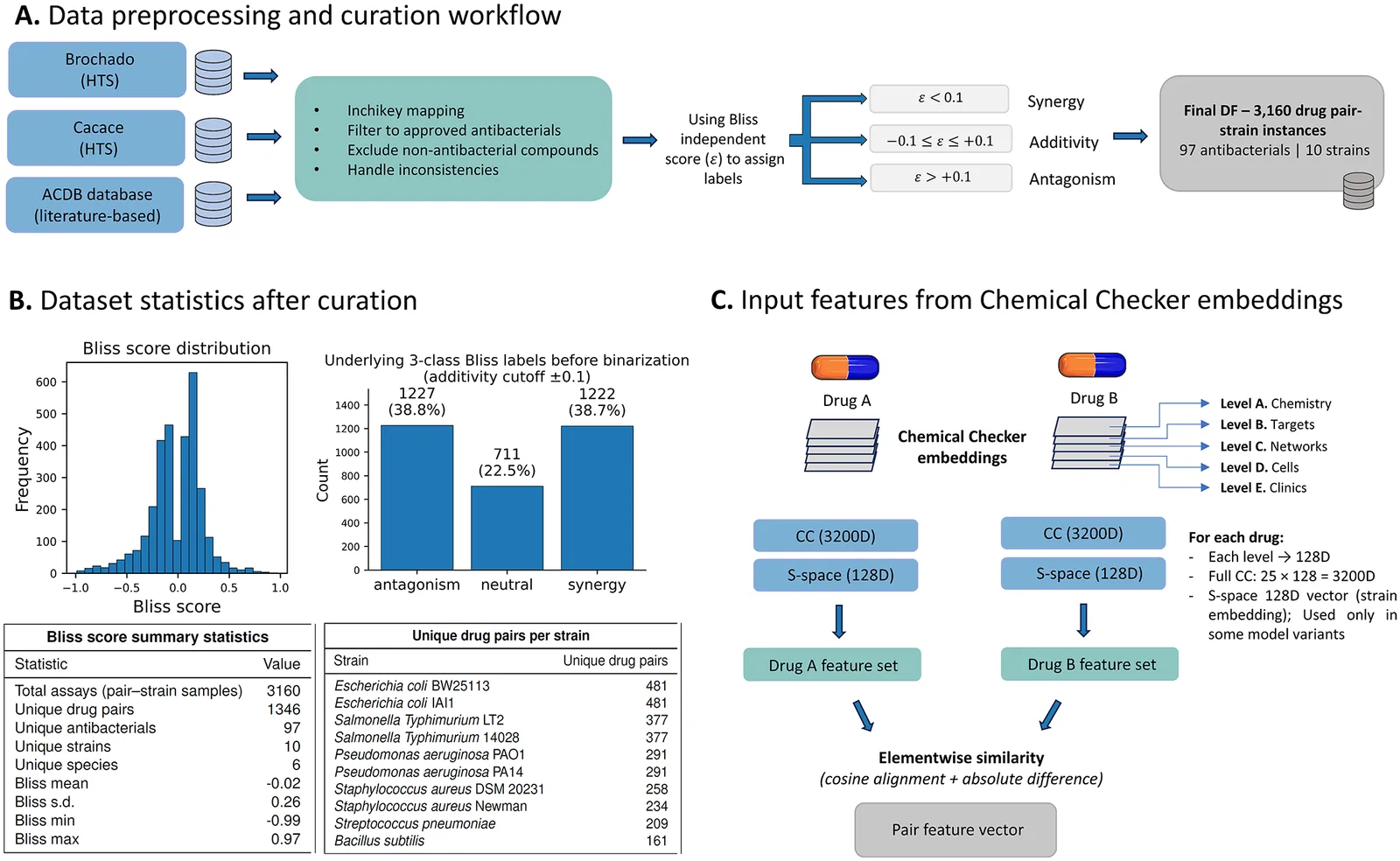
Data curation, dataset characteristics, and feature construction. (A) Workflow for preprocessing and integrating interaction data from sources. Interaction data were standardized, mapped to InChIKeys filtered to 97 antibacterials, and cleaned for duplicates/inconsistencies. Bliss labels were assigned using ε <−0.1 (synergy) and ε > +0.1 (antagonism), yielding 3,160 pair–strain measurements (2,449 binary pairs) across 10 strains. (B) Bliss score distribution, underlying 3-class labels before binarization, and summary statistics for strains, antibacterials, and score ranges. (C) Pairwise features derived from Chemical Checker embeddings: each drug’s 25 × 128-dimensional signature is converted into element-wise similarity features; an optional 128-dimensional strain embedding (S-Space) is included only in M2 and M1 models.

The final subset of 2,449 pair-strains are all synergy/antagonism binary interactions, spanning diverse drug classes and both Gram-positive and Gram-negative species (Fig 1B). The 97 compounds span two broad categories. Fifty-seven belong to conventional antibacterial classes across 25 mechanistic categories, including beta-lactams, macrolides, aminoglycosides, and fluoroquinolones (S6 Table). The remaining 40 are non-traditional antibacterial agents: compounds originally developed for other indications (e.g., NSAIDs, antipsychotics, antineoplastics) with independently documented direct antibacterial activity, reflecting the growing literature on repurposed drugs as antimicrobials. Compounds acting only as potentiators without direct antibacterial activity, such as beta-lactamase inhibitors, were excluded during curation. In our model generation discussed in the following sections, both categories are represented across training and test partitions in every outer fold, limiting the potential for class-level chemical-space bias.

The resulting binary training set was near-balanced (1,222 synergy vs. 1,227 antagonism instances, 49.9% vs. 50.1%; Fig 1B), so no explicit class-re-weighting or re-sampling was applied during model training. Each compound was then encoded using multi-level Chemical Checker bioactivity signatures [22] to generate pairwise similarity features (Fig 1C), providing a consistent representation of chemical, biological, and phenotypic drug properties. Together, the curated dataset and CC-derived features establish a standardized foundation for evaluating antibacterial synergy.

### 2.2 Constructing predictive models of drug synergy

Using the curated dataset described above, we next set out to build robust predictive models of drug-drug synergy. We first examined how different evaluation strategies affect reported performance in antibacterial synergy prediction tasks. Many existing studies evaluate models using standard stratified cross validation, in which the same drug pairs may appear in both training and test folds. This setup can unintentionally introduce information leakage, as models may effectively memorize pair-specific signals rather than learning transferable features. To systematically assess this effect, we compared several model variants under increasingly stringent evaluation schemes, and selected the most robust one as our final model named HALO (*Held-out Antibacterial interaction Learning from latent bioactivity Observations)*. Fig 2A lays out the model configurations and evaluation setups compared in this section; Panels 2B and 2C then report how these choices affect predictive performance under increasing levels of CV strictness.

**Fig 2:**
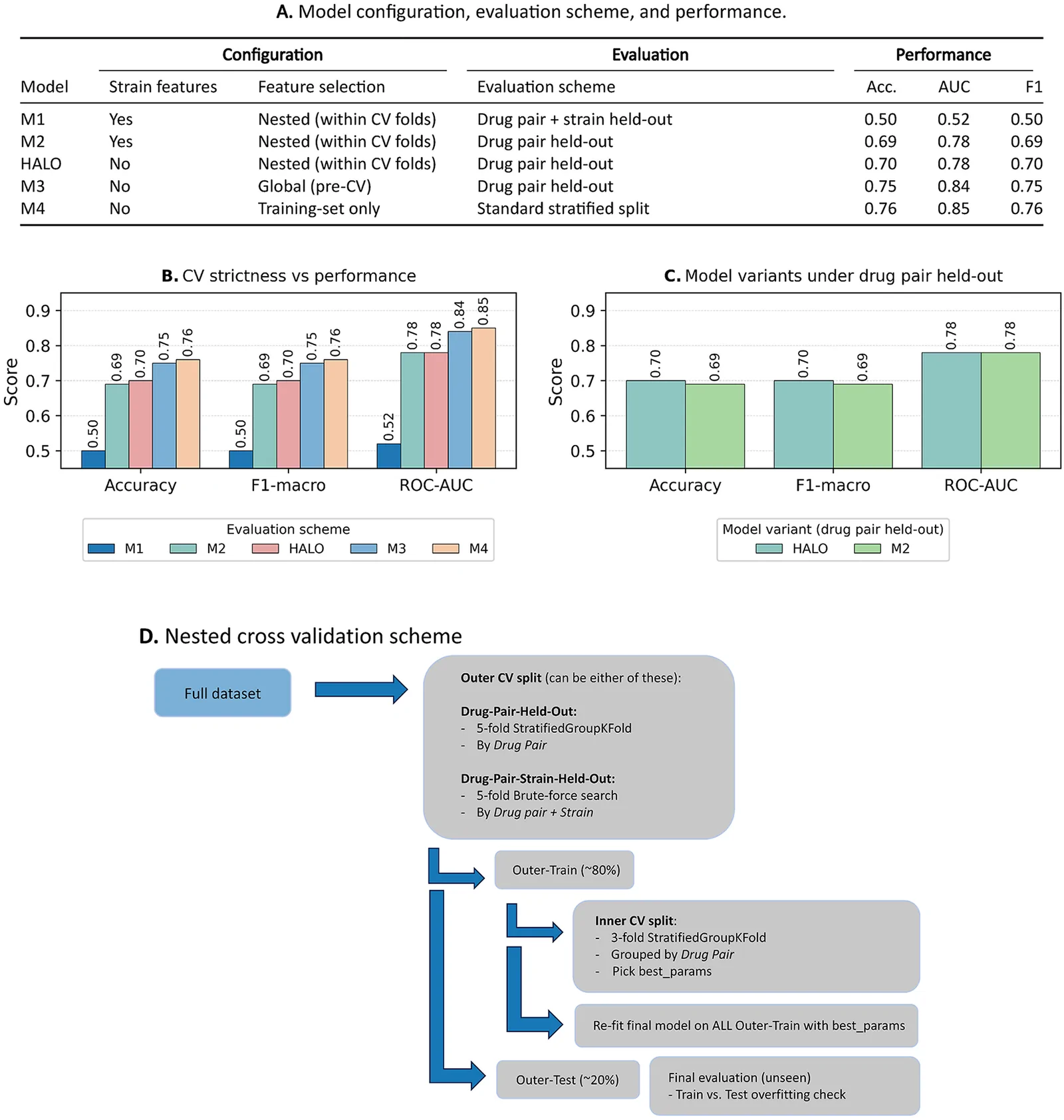
Model configuration and comparison of validation schemes. (A) Configuration and performance of the five model variants evaluated in this study. Each model differs in feature composition, feature selection strategy, and cross validation scheme. Panel A table summarizes the evaluation protocol used by each variant; standard stratified, drug-pair-heldout, or drug-pair-strain-heldout cross validations and reports the resulting performance metrics. (B) Performance of the model variants under three evaluation schemes: standard stratified CV (M4), drug-pair-held-out CV (HALO, M2, M3), and drug-pair-strain-held-out CV (M1). (C) Comparison of HALO (CC features only) vs. M2 (CC + strain-space) under drug pair held-out. (D) HALO’s nested CV workflow: a 5-fold outer split (drug-pair-held-out or drug-pair-strain-held-out) defines held-out data; a 3-fold inner CV grouped by drug pair tunes hyperparameters; the final model is refit on the outer-train fold and evaluated once on the outer-test fold.

With the exception of M4, all models use a nested cross validation procedure consisting of a 5-fold outer split (either with drug-pair-held-out or drug-pair-strain-held-out) and a 3-fold inner loop grouped by drug pair for hyperparameter selection (Fig 2D). Under standard stratified CV (M4), where drug pairs can appear in multiple folds, performance was substantially inflated (ROC–AUC = 0.85; accuracy/F1-macro/F1-weighted = 0.76; one-sample t-test against HALO’s outer-fold distribution, all p ≤ 0.011; S1 Table). A partially drug-pair-held-out scheme in which feature selection was performed globally prior to cross validation (M3) produced similarly optimistic results (ROC–AUC = 0.84; accuracy/F1-macro = 0.75; paired t-test vs. HALO, all p = 0.002; S1 Table), illustrating that performing preprocessing outside the cross-validation loop can also introduce leakage. To directly quantify this feature-selection leakage, we compared the composition of M3’s globally selected feature set against HALO’s five independently selected fold-internal sets. M3 selected an essentially fixed set of 1,600 features in every outer fold (mean pairwise Jaccard overlap = 1.000 across all fold pairs), since selection was performed once on the full dataset prior to splitting. HALO’s fold-internal selection yielded a comparable mean feature count (1,609 ± 7 per fold; paired t-test on count, p = 0.033) but substantially lower fold-to-fold similarity (mean pairwise Jaccard = 0.296 ± 0.009), reflecting genuine adaptation to each fold’s available training data rather than reuse of a single globally optimized set. While approximately half of M3’s global feature set (47–50%) overlapped with any individual HALO fold, 226 features (14.1% of M3’s global set) were never selected in any of the five HALO folds despite being available in the same underlying feature pool. These features are only accessible to M3’s model because its selection procedure had visibility into the complete dataset, including instances later held out for testing — providing direct, feature-level evidence for the leakage mechanism underlying M3’s inflated performance (S2 Table). When pair-level separation was enforced and feature selection was performed independently within each fold (M2 and HALO), performance decreased to 0.70 accuracy/F1-macro/F1-weighted and 0.78 ROC–AUC, and the difference between M2 and HALO was not statistically significant on any metric (paired t-test, all p ≥ 0.26), indicating that strain-space features did not provide detectable additional signal under this evaluation scheme. Finally, the most stringent evaluation scheme held out both drug pairs and bacterial strains (M1). Under this regime, performance dropped to near-random levels (accuracy = 0.51 ± 0.01, ROC–AUC = 0.53 ± 0.03 across folds) highlighting the difficulty of predicting simultaneously across unseen drug-pair and strains. Notably, this reduction in performance primarily reflects data sparsity rather than model failure: drug pair coverage across strains was limited. While 31% of pairs were observed in only a single strain, 40% in two strains, and only a small fraction was measured across multiple strains. Consequently, many pair–strain combinations in the heldout folds correspond to drug pairs never observed in any other strain, preventing the model from learning strain-specific interaction patterns. Under these conditions, explicit strain embeddings add little beyond Chemical Checker–derived bioactivity similarities, which already captures much of the transferable mechanistic signal. Consistent with this interpretation, Fig 2C shows that the strain-aware model variant (M2) performs almost identically to HALO when evaluated under drug-pair held-out cross validation, indicating that CC-derived similarity features capture most of the predictive signal available under this setting. Fig 2D summarizes the nested CV workflow: the outer split defines the held-out fold, the inner loop selects hyperparameters, and the final model is refit on the full outer-training set before a single evaluation on the untouched outer-test fold. M4 lacks this safeguard and therefore overestimates performance. Overall, these comparisons demonstrate that evaluation protocol strongly influences reported performance. Permissive validation schemes substantially overestimate accuracy, whereas strict drug-pair held-out evaluation provides a more stable and realistic benchmark for antibacterial synergy prediction. HALO therefore adopts this leakage-aware evaluation framework to assess generalization to unseen drug combinations.

Beyond evaluation protocol, the choice of prediction task itself (binary, multi-class, or regression) also shapes how reliably interaction outcomes can be modeled. Before finalizing our modeling approach, we compared binary classification against two alternative task formulations using the same feature set and CV scheme (S3 Table): three-class classification (synergy/additive/antagonism) and regression on continuous Bliss ε. Three-class classification underperformed substantially relative to the binary framing (accuracy 0.52, F1-macro 0.45, versus 0.70 and 0.70 for HALO), and the larger drop in F1-macro relative to F1-weighted (0.45 vs. 0.50) indicates that performance was disproportionately dragged down by the harder-to-resolve class. This is consistent with the additive class being the most label-uncertain, as discussed below. Regression on continuous ε performed comparably poorly (R^2^ = 0.25, MAE = 0.17), explaining only a quarter of the variance in interaction strength. Notably, the regression model’s average error (MAE = 0.17) exceeds the ± 0.1 margin used to define the neutral/additive band, meaning that even continuous predictions could not reliably resolve which side of the additivity boundary a given interaction truly falls on. Regression therefore does not avoid the boundary-uncertainty problem discussed below. Rather, it defers it to an unmodeled downstream threshold, while also being harder to translate into a binary clinical decision. Based on these comparative analyses, we adopted binary classification of clear synergy versus antagonism as HALO’s primary task.

### 2.3 Model performance evaluation

We developed HALO using a LightGBM classifier trained on pairwise similarity features derived from Chemical Checker embeddings. Starting from 6,400 raw features representing element-wise similarities across multiple bioactivity domains, the model was evaluated under a strict 5-fold drug-pair heldout nested cross validation framework. In each outer fold, all interactions corresponding to approximately 20% of drug pairs were withheld for testing. Hyperparameter tuning was performed within the training portion of each split using a nested 3fold grouped cross validation procedure. To avoid information leakage, feature selection was embedded within this nested workflow rather than performed as a separate preprocessing step. Features were therefore selected using only the training data within each fold, and the final model was refit on the full outer training set before evaluation on the untouched outer test fold. As a result, the exact subset of selected features could vary across folds.

Under this pair-held-out setting, HALO achieved 0.70 accuracy, F1-macro, and F1-weighted, and a ROC–AUC of 0.78 on the held-out data (Fig 3A–3D). The confusion matrix showed balanced performance across synergy and antagonism, with comparable false-positive and false-negative rates (Fig 3B). Performance also remained broadly consistent across folds (Fig 3E), indicating reproducible generalization to unseen antibacterial combinations under strict pair-level separation. Importantly, our contribution lies not in introducing a novel molecular representation, but rather in combining multilevel bioactivity signatures with a leakage-aware evaluation framework that distinguishes drug-pair memorization from transferable biological similarity. This approach yields a more conservative but biologically meaningful benchmark for antibacterial synergy prediction. A sensitivity analysis of the Bliss neutrality cutoff was included to confirm that the HALO model performance was robust to threshold choices (S1 Fig).

**Fig 3:**
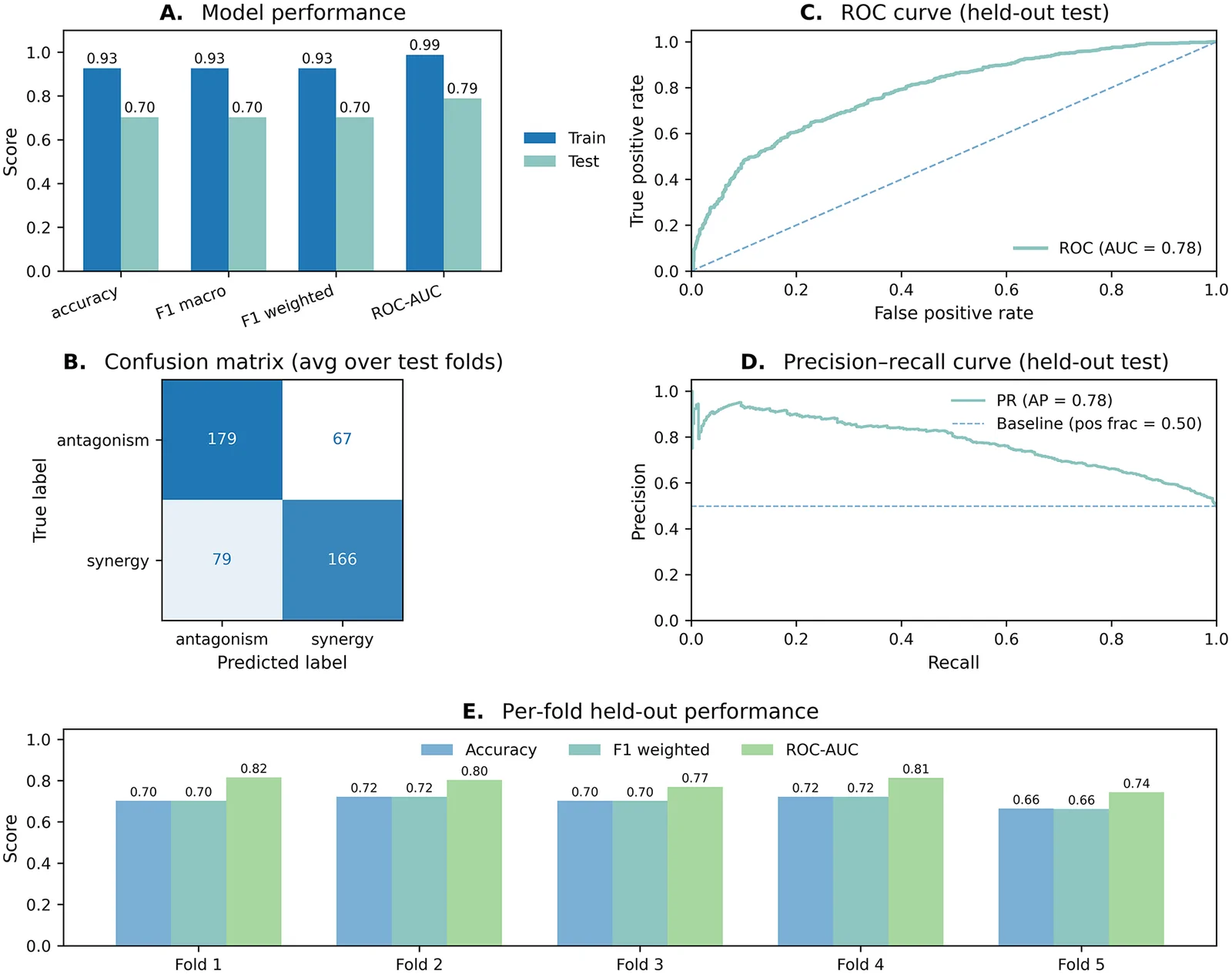
HALO’s performance under drug-pair-held-out cross-validation. (A) Train and outer-test performance (accuracy, F1-macro, F1-weighted, ROC-AUC) in HALO, where all drug pairs in the test fold are unseen. Test metrics range ~0.70–0.79. (B) Average confusion matrix across outer folds (balanced classes). (C) ROC curve for held-out predictions (AUC = 0.78). The dotted line indicates random-chance performance. (D) Precision–recall curve (AP = 0.78; baseline = 0.50). (E) Per-fold outer-test accuracy, F1-weighted, and ROC-AUC, showing stable performance across all five folds (≈0.66–0.82).

### 2.4 Insights from Chemical Checker embeddings

Next, we examined which Chemical Checker feature groups contributed most strongly to synergy prediction. We quantified feature importance from the trained HALO models using gain-based LightGBM importance, which captures each feature’s contribution to loss reduction across decision-tree splits (Fig 4A–4C).

**Fig 4:**
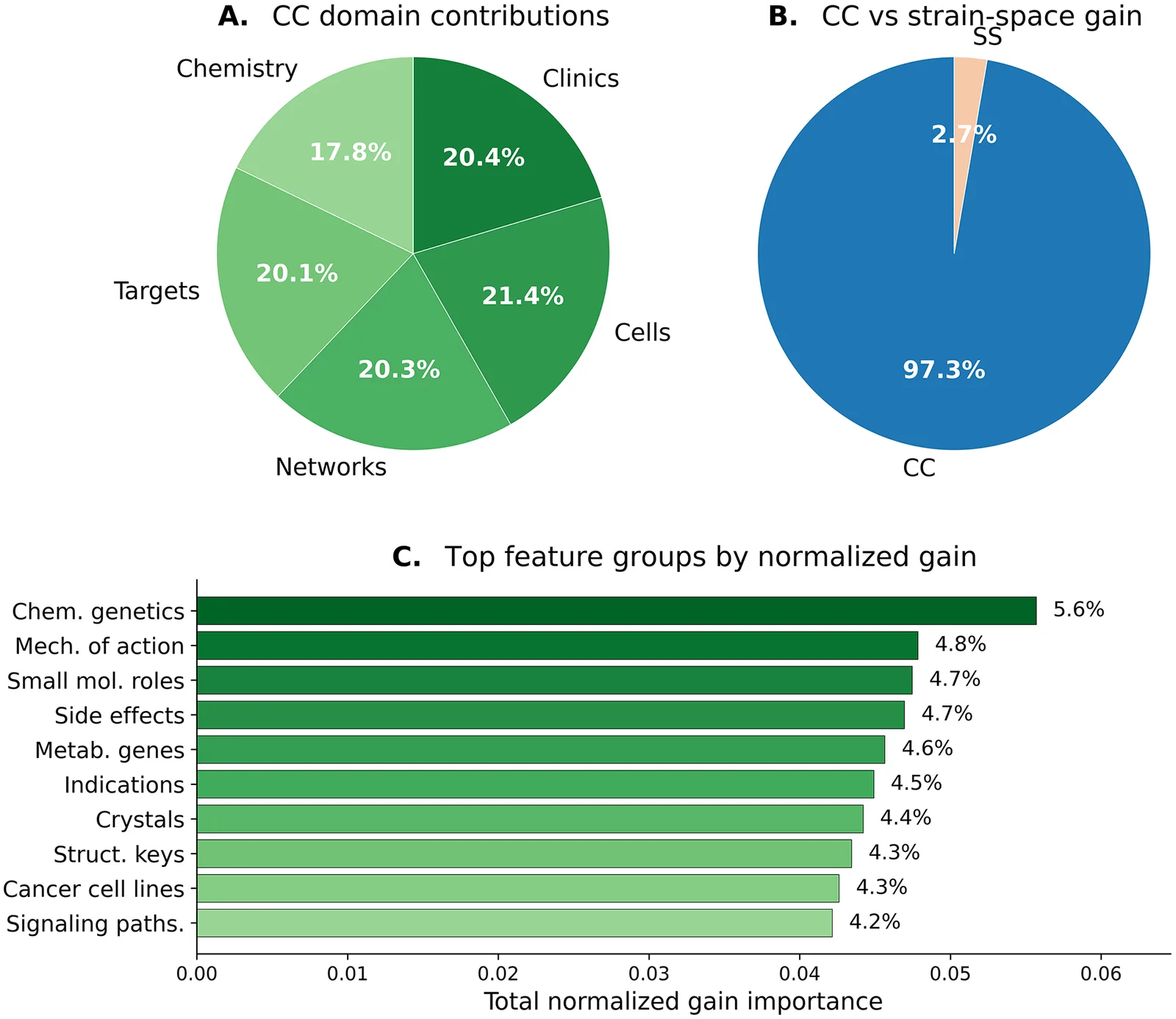
Feature contributions to HALO model. (A) Total gain contributions from the five CC domains (Networks, Cells, Clinics, Targets, Chemistry). (B) Gain comparison in the strain-aware variant (M2): CC features account for 97.3% of total gain, S-space for 2.7%. (C) Normalized gain aggregated across the most informative Chemical Checker (CC) sub-levels.

At the five top-level CC domains, contributions were nearly uniform (~17–21% each; Fig 4A), indicating that synergy prediction draws from a broad, multi-scale bioactivity representation rather than any single biological layer, for example, structural information. Adding strain-space embeddings had little effect: in M2, CC features accounted for 97.3% of total gain, whereas strain-space contributed only 2.7% (Fig 4B).

CC features showed broadly distributed importance, with several chemical-genetic and phenotypic dimensions ranking as the highests. To interpret these signals at higher levels of biological organization, we aggregated importance across the Chemical Checker hierarchy (Fig 4C). At the sub-level scale chemical-genetics, mechanism of action, small-molecule roles, side-effect profiles and metabolic genes accounted for the largest cumulative gain. Overall, synergy prediction is supported by diverse CC bioactivity modalities, and CC signatures; not this current strain embeddings, and that the HALO model’s interpretability is driven by a consistent pattern of biologically meaningful CC sub-levels.

To verify that the gain-based importance patterns were not an artifact of the LightGBM-specific importance metric, we additionally computed SHAP values for the final model. The SHAP-based feature ranking was positively correlated with the gain-based importance ranking (Spearman ρ = 0.66, p = 1.1 × 10^-179^), confirming a non-random association. Importantly, while the precise ordering of individual features differed — as expected given the fundamental differences between these two importance metrics — both approaches consistently identified the same high-level Chemical Checker sub-levels (e.g., chemical genetics, mechanisms of action, small-molecule roles, and side-effect profiles) among the most predictive signals. This convergence across orthogonal interpretation methods supports the biological robustness of the CC-derived feature representation underlying HALO’s predictions

### 2.5 External validation and algorithm benchmarking

Next, to assess generalization beyond our initial training dataset, we evaluated the model on an independent dataset. We tested HALO on an external interaction dataset from Chandrasekaran et al. (INDIGO) [17], which differs in assay type, interaction scoring, and drug–pair composition. INDIGO reports Loewe α interaction scores for diverse antibiotic pairs in *E. coli* MG1655 that are calculated differently compared with Bliss ε. Rather than converting Loewe α to Bliss ε on a continuous scale, we adopted the synergy/antagonism thresholds defined in the INDIGO study itself (α < –0.5 synergy; α > 1 antagonism), which was described as approximately corresponding to strong Bliss-like deviations from additivity [17]. The Loewe method assumes that the line of constant growth is linear for additive drug pairs and synergy or antagonism are concavity or convexity deviations of the growth contour. Panel 5A summarizes the preprocessing pipeline used to map INDIGO compounds to InChIKeys, filter to approved antibacterials, attach CC similarity features, and assign synergy/antagonism labels from Loewe α scores. Because INDIGO and HALO’s training data are drawn from partially overlapping antibacterial interaction literature, we additionally checked for drug-pair overlap at the InChIKey level between the INDIGO set and our internal training data, independent of strain. Of the 70 curated INDIGO pairs, the 36 (51.4%) that shared a drug pair already present in our training set were excluded, leaving 34 pairs representing genuinely unseen drug combinations for external evaluation. On this non-overlapping subset, HALO achieved a ROC-AUC of 0.90 (Fig 5B), despite being trained only on Bliss-based interactions from different strains, indicating that HALO’s external performance is not driven by pair-level memorization. Given the small sample size (n = 34) and limited number of true synergy cases (6 of 34), threshold-dependent metrics were more variable: Average precision was 0.76, well above the 0.18 positive-class baseline (Fig 5C). Accuracy reached 0.74 and synergy-class F1 was 0.57, driven by perfect synergy recall (6/6) alongside reduced precision (9 false positives among predicted synergy calls; Fig 5D). This external set was class-imbalanced (18% synergy after filtering; Fig 5C), unlike our near-balanced internal training data (Fig 1B) — a natural consequence of the independent dataset’s own interaction distribution rather than a property we controlled for. We therefore report PR-AUC/average precision alongside ROC-AUC here specifically, as these are more informative than accuracy or ROC-AUC alone under class imbalance. These results show that HALO learns a transferable mapping from CC-derived similarity to antibacterial interaction outcomes, generalizable across assay types, strains, and data sources.

**Fig 5:**
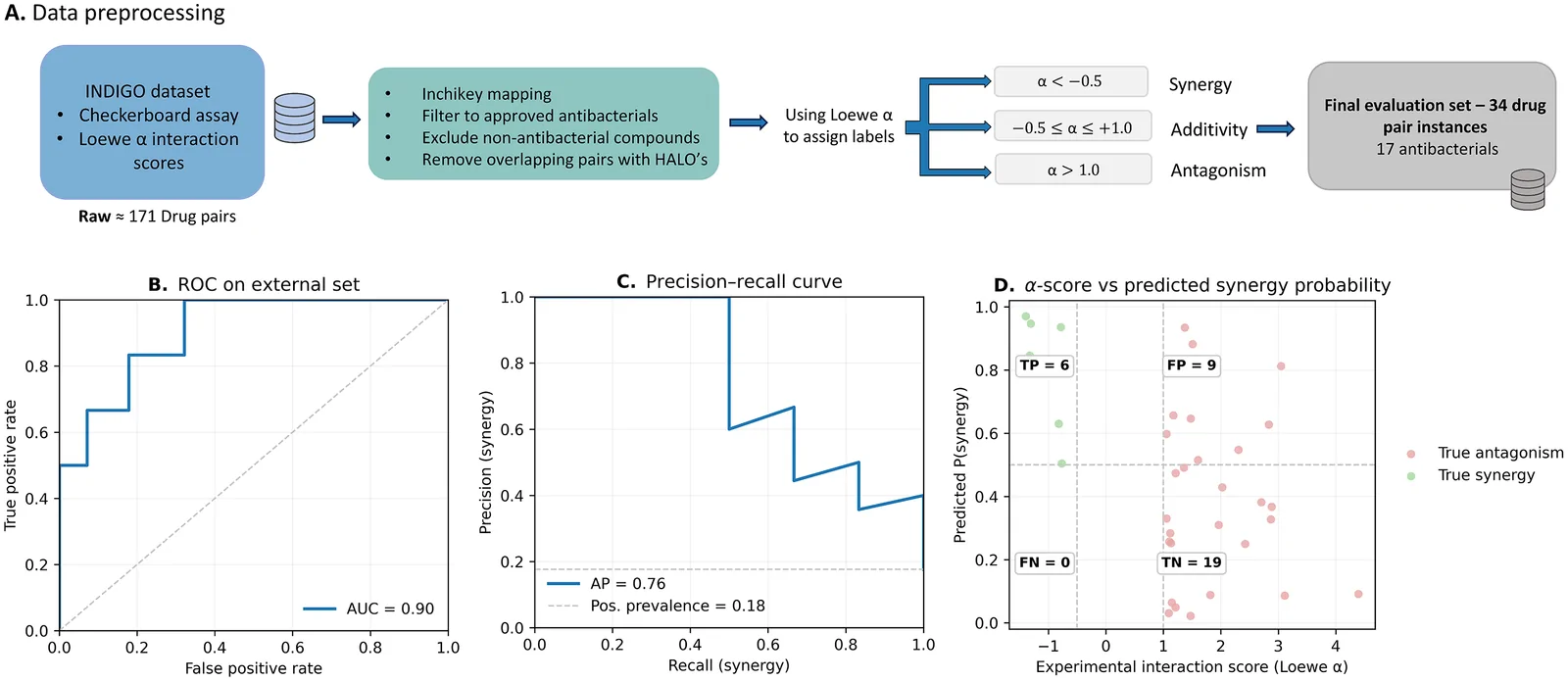
External validation on the INDIGO dataset. (A) External data preprocessing. The INDIGO dataset (≈171 drug pairs, *E. coli* MG1655) was mapped to InChIKeys, filtered to approved antibacterials, and pairs overlapping HALO’s training data were removed. Loewe α thresholds (α <−0.5 synergy; −0.5 ≤ α ≤ +1.0 additivity; α > 1.0 antagonism) defined labels, yielding a final evaluation set of 34 drug-pair instances across 17 antibacterials. (B) ROC curve for HALO on the 34 non-overlapping INDIGO pairs (AUC = 0.90). (C) Precision–recall curve (AP = 0.76; positive-class prevalence = 0.18). (D) Experimental Loewe α interaction scores versus predicted P(synergy); dashed lines indicate the α classification thresholds (left) and the 0.5 decision boundary (bottom). Confusion outcomes: TP = 6, FP = 9, FN = 0, TN = 19.

As a second, independent external validation, we next tested a subset of the Antibiotic Combination Database (ACDB) [27] that was not used during HALO’s training. While ACDB Bliss-scored interactions were incorporated into our primary training dataset (Section 3.1), a separate subset of ACDB reports interactions scored using the FICI or Loewe methods rather than Bliss independence; these rows were never used for HALO’s training and were processed through the same curation pipeline as our primary dataset and the INDIGO validation set. After filtering to approved antibacterials and mapping to InChIKeys, this subset comprised 334 pair-strain instances. Following the same drug-pair overlap check applied to INDIGO, 125 of these rows shared a drug pair with our training data and were excluded. After further restricting to clear synergy/antagonism outcomes (excluding additive interactions, consistent with Section 3.2), 76 pair-strain instances remained (71 scored via FICI, 5 via Loewe). HALO achieved a ROC-AUC of 0.71 and F1-macro of 0.55 on this set (Fig 6). This external cohort was strongly synergy-skewed (66 synergy vs. 10 antagonism instances), the converse imbalance from the INDIGO validation set. Under this distribution, HALO showed strong synergy recall (42/66) alongside reduced antagonism precision, correctly identifying 7 of 10 true antagonistic pairs. Together, external validation on two independent datasets — INDIGO (Loewe-scored, antagonism-skewed) and ACDB (FICI/Loewe-scored, synergy-skewed) — demonstrates that HALO generalizes across differing assay types, scoring methodologies, and class distributions, with both validations restricted to drug pairs entirely absent from training.

**Fig 6:**
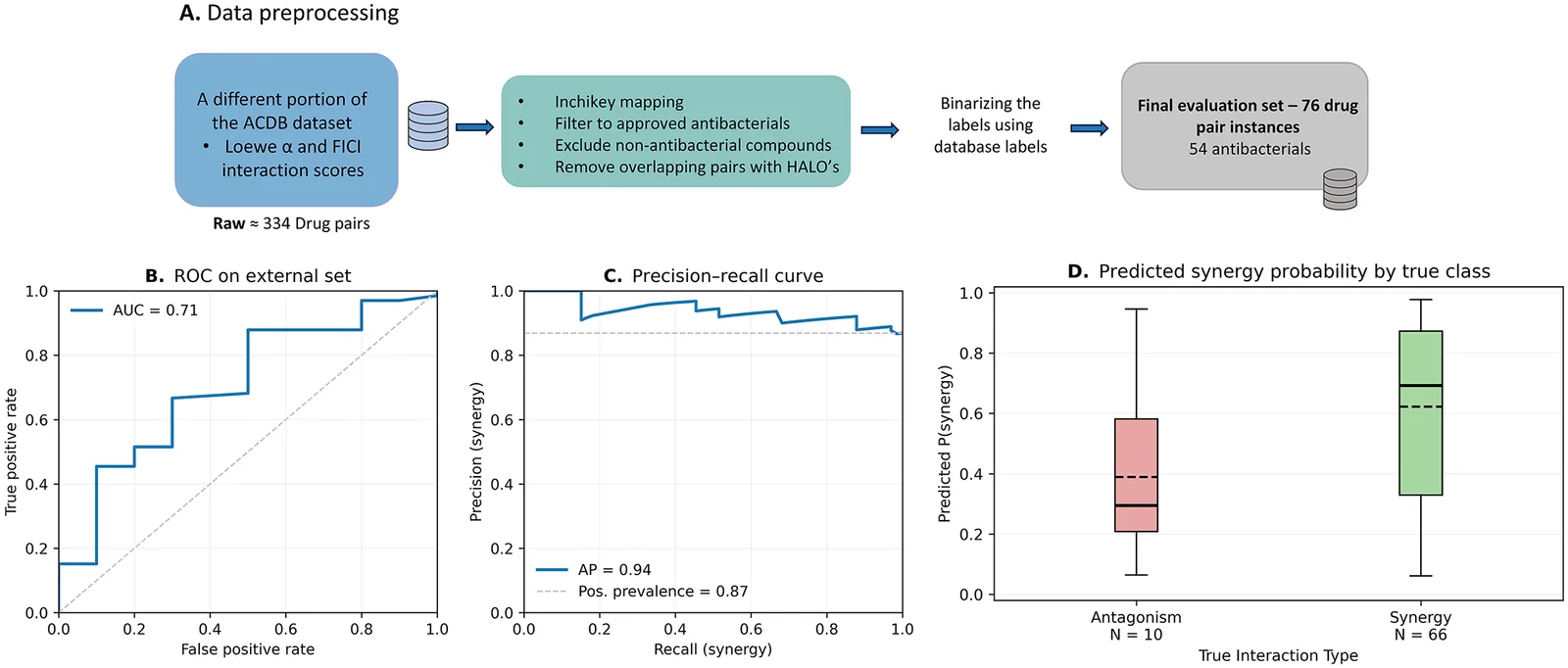
External validation on an independent ACDB subset. (A) External data preprocessing. A portion of the Antibiotic Combination Database (ACDB) not used in HALO’s training — comprising interactions scored via Loewe α or FICI (≈334 drug pairs) — was mapped to InChIKeys, filtered to approved antibacterials, and pairs overlapping HALO’s training data were removed. Labels were binarized using each interaction’s reported database classification, yielding a final evaluation set of 76 drug-pair instances across 54 antibacterials. (B) ROC curve for HALO on this set (AUC = 0.71). (C) Precision–recall curve (AP = 0.94; positive-class prevalence = 0.87). (D) Distribution of predicted P(synergy) by true interaction class (solid line = median, dashed line = mean); true antagonism, N = 10; true synergy, N = 66.

To provide a direct, same-dataset benchmarking comparison against feature representations comparable in scope to CoSynE and INDIGO, we retrained HALO’s pipeline (identical nested pair-held-out cross-validation protocol), this time by restricting the feature set to a single CC sub-level resembling each method’s original representation (A4 structural key features for CoSynE; D3 chemical genetics features for INDIGO) and replacing LightGBM with a Random Forest classifier, matching the algorithm family used by both prior frameworks. Both narrow-feature RF variants underperformed HALO’s full multi-level model (Fig 7): RF (CoSynE-style) achieved accuracy = 0.66 ± 0.02 and ROC-AUC = 0.72 ± 0.03, and RF (INDIGO-style) achieved accuracy = 0.65 ± 0.01 and ROC-AUC = 0.72 ± 0.01 (mean ± SD, five outer folds), compared to HALO’s accuracy = 0.70 ± 0.02 and ROC-AUC = 0.78 ± 0.03. Taken together, these results support HALO’s full multi-level CC representation over narrower, single-sublevel feature sets resembling prior antibacterial-specific frameworks, most clearly under our own strict internal evaluation protocol.

**Fig 7:**
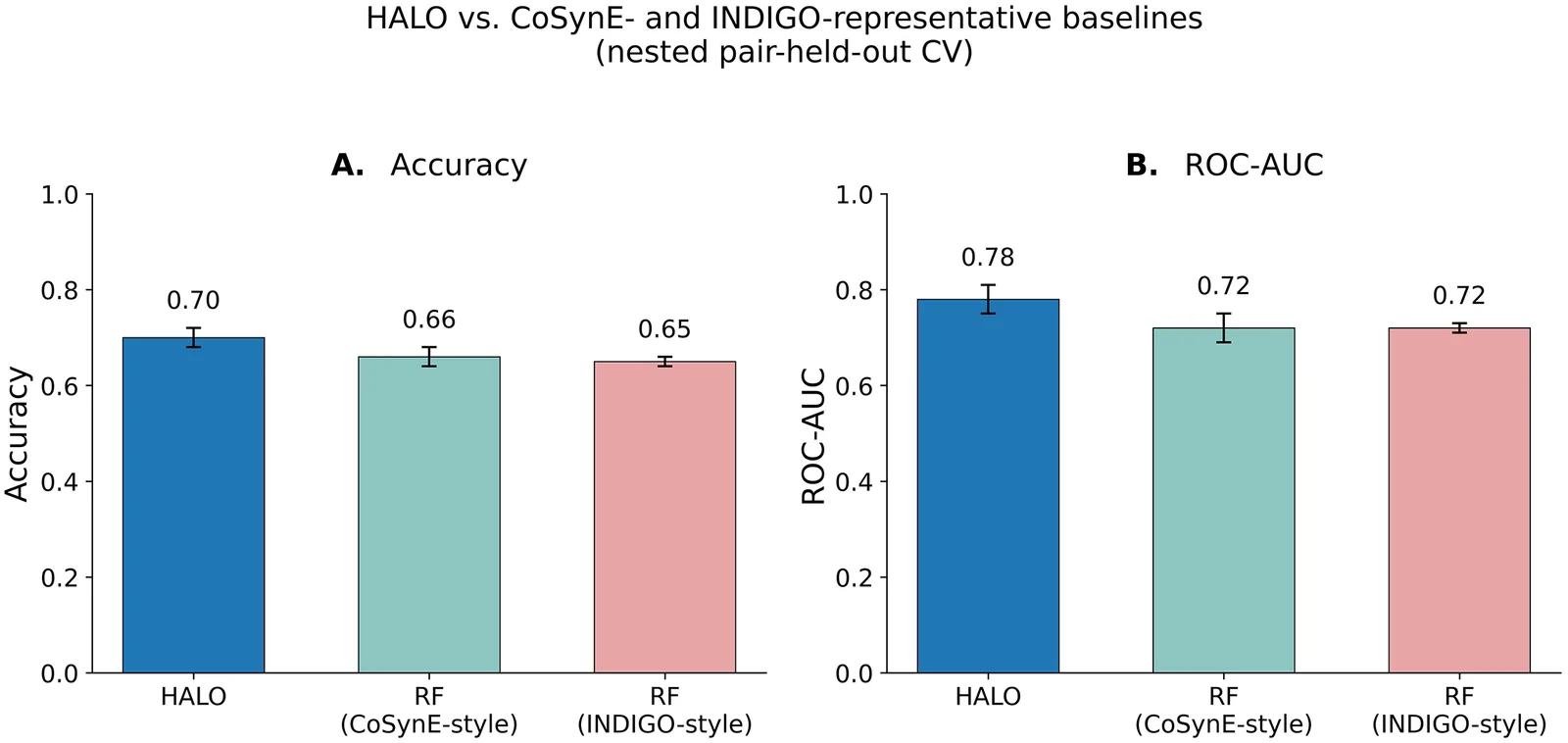
Benchmarking against CoSynE- and INDIGO-representative baselines. HALO’s full multi-level Chemical Checker representation was compared against two narrow-feature Random Forest baselines under identical nested pair-held-out cross-validation: one restricted to CC sublevel A4 (structural keys), resembling CoSynE’s structural fingerprint representation, and one restricted to CC sublevel D3 (chemical genetics), resembling INDIGO’s chemogenomic representation. **(A)** Accuracy and **(B)** ROC-AUC, mean ± SD across five outer folds. HALO outperformed both narrow-feature baselines on both metrics.

### 2.6 Novel drug-pair candidates

Using the final HALO model trained on all labeled interactions, we generated predictions for all drug pairs absent from the training set. Full ranked lists of synergistic and antagonistic pairs are provided in S4–S5 Tables.

We next assessed high-confidence predictions in further detail. Table 2 shows top synergy candidates predicted by the HALO. Several of these top predictions align with known mechanisms. The novel combinations identified by our model do not appear arbitrary; rather, they converge on a small number of biologically coherent synergy archetypes that are already established in the antibacterial literature. The largest cluster of novel predictions — comprising five of the ten highest-confidence pairs — consists of cephalosporin–penicillin dual β-lactam combinations: Cefaclor + Penicillin V, Cefaclor + Temocillin, Cefaclor + Ticarcillin, Cefaclor + Mezlocillin, and Carbenicillin + Cefaclor. Although none of these specific pairings has been formally tested for synergy, their predicted activity is mechanistically grounded in one of the best-characterised phenomena in antibacterial combination therapy. β-Lactam antibiotics display heterogeneous and drug-specific penicillin-binding protein (PBP) profiles that catalyze the final cross-linking steps of peptidoglycan assembly [33]. This selectivity can produce complementary blockade of peptidoglycan synthesis when two β-lactams are combined. The most defensible interpretation of the cefaclor-containing pairs is therefore that they extend the classical logic of sequential or complementary cell-wall inhibition, in which one drug strengthens the functional impact of the other by narrowing the bacterium’s ability to compensate at the level of PBPs and cell-wall remodeling [34].

**Table 2:** Top 10 predicted synergistic pairs generated by the HALO-CV1 model, ranked by predicted probability P(syn).

Drug Pair	*P*(syn)	*P*(ant)	Drug Classes
Cefaclor + Penicillin V	0.999	0.000	Cephalosporin (2nd gen) – Penicillin
Bacitracin + Capreomycin	0.999	0.000	Polypeptide antibiotic – Aminoglycoside antibiotic
Cefaclor + Temocillin	0.998	0.001	Cephalosporin (2nd gen) – Penicillin
Benzylpenicillin + Flucloxacillin	0.998	0.001	Penicillin – Penicillin
Cefaclor + Ticarcillin	0.998	0.001	Cephalosporin (2nd gen) – Penicillin
Bacitracin + Vancomycin	0.998	0.001	Polypeptide antibiotic – Glycopeptide antibiotic
Cefaclor + Mezlocillin	0.997	0.002	Cephalosporin (2nd gen) – Penicillin (ureidopenicillin)
Carbenicillin + Cefaclor	0.997	0.002	Penicillin (carboxypenicillin) – Cephalosporin (2nd gen)
Chir-90 + Tylvalosin	0.997	0.002	Lipid A biosynthesis inhibitor – Macrolide antibiotic
Colistin A + Colistin B	0.997	0.002	Polymyxin antibiotic – Polymyxin antibiotic

This same logic also explains why the model repeatedly converged on envelope-active combinations. Polymyxins have a well-established role as membrane-permeabilizing agents, and their synergy with other antibiotics is widely attributed to increased uptake and deeper access of the partner drug into the cell [35]. CHIR-090 provides an especially compelling analogue for that pattern because it inhibits lipid A biosynthesis, weakening the outer membrane at an upstream step [36] that is mechanistically compatible with a second hit on protein synthesis, such as tylvalosin. Tylvalosin, like all macrolides, acts by binding to the peptidyl transferase region of the bacterial 50S ribosomal subunit and blocking translocation [37]. Its ribosomal target is universally conserved across both Gram-positive and Gram-negative bacteria, and studies using hyperpermeable *E. coli* mutants have confirmed that macrolides retain full target engagement in Gram-negative cells when the outer membrane barrier is removed [38]. The problem, therefore, is not affinity but access: the Gram-negative outer membrane, whose structural integrity depends almost entirely on the lipid A anchor of lipopolysaccharide (LPS), excludes bulky hydrophobic molecules such as macrolides with high fidelity [39]. CHIR-90 attacks this barrier at its biosynthetic root, inhibiting LpxC — the zinc-dependent deacetylase that catalyses the committed step of lipid A synthesis — and thereby progressively dismantling the outer membrane from within [40]. Published in vitro checkerboard experiments have demonstrated that LpxC inhibitors synergise with rifampicin, vancomycin, tetracycline, and other antibiotics whose activity against Gram-negative organisms is constrained by outer membrane impermeability [41], and a companion mechanism study showed that a structurally related LpxC inhibitor enhanced the antibacterial activity of meropenem against carbapenem-resistant Enterobacteriaceae by increasing membrane permeability and facilitating intracellular drug entry [42]. Taken together, the mechanistic logic is clear even though the exact combination has not been previously reported as synergistic: CHIR-90 serves as a molecular key that breaches the outer membrane, transforming Tylvalosin from a Gram-positive-spectrum agent into a broad-spectrum one by granting it access to a ribosomal target it can already bind with high affinity. This pairing therefore represents a mechanistically plausible computational hypothesis — one that mirrors an emerging therapeutic paradigm in which outer membrane-disrupting agents are deliberately paired with antibiotics that have historically been prescribed for Gram-negative infections — though of course experimental validation will be required to confirm synergy for these candidate combinations.

Similar observations were made on other envelope-focused pairs. Bacitracin + Vancomycin and Bacitracin + Capreomycin are two pairs that exploit a different but equally well-characterized vulnerability: Bacitracin and vancomycin attack different stages of cell-envelope construction, so their combination can be interpreted as coordinated pressure on precursor recycling and peptidoglycan assembly rather than simple target duplication. Even where the exact pair was not previously reported, the broader literature supports the principle that dual interference with envelope homeostasis is frequently more effective than either agent alone, especially when the second drug exploits the vulnerability created by the first. A published study of non-β-lactam antibiotics against *Enterococcus hirae* provided direct mechanistic confirmation, showing that bacitracin in combination with penicillin produced a synergistic effect associated with a marked reduction in PBP density — and proposing that this effect stems from the inhibition of PBP synthesis by drugs acting on early stages of peptidoglycan synthesis, including the bactoprenol cycle [43].

For these reasons, the model’s novel pairs are not best viewed as isolated predictions; they are better understood as new instances of established pharmacological logic, in which structurally related or mechanistically complementary antibiotics create a higher-order effect that is already familiar from prior synergy studies. We also emphasize that these remain computational hypotheses generated from bioactivity-based pattern recognition rather than experimentally confirmed interactions; experimental validation (e.g., checkerboard assays) is required before any of these pairs can be considered candidates for further pharmacological development.

## 3. Methods

### 3.1 Data sources and antibacterial curation

Interaction data were compiled from three sources: high-throughput checkerboard assays from Brochado et al. [9], Cacace et al. [10], and manually curated pairwise interaction entries from the ACDB database [29]. To define a consistent antibacterial compound space, we constructed a unified list of antibacterial compounds by integrating FDA-approved agents with research and literature-reported antibacterial compounds. Non-antibacterial agents, combination products, and β-lactamase inhibitors were excluded. All compound identities were standardized by mapping names and stereochemical variants to canonical InChIKeys. The dataset spans 10 commonly used Gram-positive and Gram-negative strains, including *E. coli* BW25113/IAI1, *S. enterica Typhimurium* 14028/LT2, *P. aeruginosa* PA14/PAO1, *S. aureus* DSM20231/Newman, *S. pneumoniae*, and *B. subtilis,* enabling evaluation across diverse physiological contexts.

### 3.2 Interaction-score harmonization and dataset integration

All datasets reported Bliss ε values but differed in checkerboard resolution and aggregation. After removing exact duplicates, replicated measurements for the same drug–strain pair were merged using a reproducible rule: exact replicates were collapsed; near-consistent values (|Δε| ≤ 0.05) were averaged; and conflicting sets were curated by discarding the farthest-from-median value before averaging. Rows with missing essential identifiers were excluded. Each (Drug A, Drug B, Strain) combination was represented by one harmonized ε score, yielding 3,160 interactions across 97 antibacterials and 10 strains.

To obtain reliable binary labels, we excluded interaction values close to the additive boundary. Bliss ε is a derived quantity that depends on the observed combination response and the two constituent single-drug responses; consequently, uncertainty in experimental growth measurements can propagate into ε and is expected to have the greatest impact near ε ≈ 0. This issue is amplified in our setting because the final dataset was harmonized across multiple studies, strains, assay conditions, and aggregation schemes, increasing heterogeneity around the additivity boundary. We applied a conservative margin-based exclusion rule and removed samples with |ε| ≤ 0.1 before modeling. Interactions with ε <−0.1 were labeled as synergy and ε > +0.1 as antagonism.

### 3.3 Feature set construction

For each drug, we retrieved sig2 Chemical Checker (CC) embeddings (25 × 128-dimensional subspaces). Pairwise drug features were computed using element-wise cosine similarity and Euclidean-derived similarity (1 − normalized L2 distance). Across 25 CC spaces, this produced 6,400 raw features per drug pair. Because CC embeddings are bounded and internally normalized, no additional feature scaling was applied. A strain-space embedding derived from strain–drug response profiles was also generated using the Chemical Checker protocol [44]. This additional feature set was used only in the strain-aware M2 and M1 models.

### 3.4 Model training and nested cross-validation

A LightGBM framework was used for binary classification, with “synergy” treated as the positive class. Training for M1, M2, M3 and HALO followed a nested cross-validation design to ensure strict separation between model selection and evaluation. The outer split defined the held-out test set and was constructed using one of two grouping schemes. In the drug-pair-held-out scheme, five outer folds were generated with a StratifiedGroupKFold splitter grouped by drug pair, such that all measurements for approximately 20% of drug pairs were withheld together in each fold. In the drug-pair-strain–held-out scheme, five independent brute-force outer splits were generated by selecting subsets of strains whose induced drug-pair test sets fell within predefined size bands (16–28% of all rows). For each drug-pair-strain-held-out split, both strains and drug-pair combinations present in the test subset were excluded from training, and any cross-edge rows violating this disjointness were removed. Hyperparameter tuning occurred strictly inside the training portion of each outer split using a three-fold StratifiedGroupKFold grouped by drug pair. Thirty-two LightGBM configurations were sampled from a predefined search space, candidate inner-loop models were trained with early stopping for stable convergence, and configurations were ranked by mean inner-CV accuracy. Across outer folds, the selected LightGBM configurations were consistently shallow and strongly regularized, with max_depth = 3, num_leaves = 7–9, learning_rate = 0.02–0.03, min_data_in_leaf = 200–300, and moderate feature/bagging subsampling with L2 regularization.

To reduce the high-dimensional elementwise similarity representation prior to model fitting, a LightGBM-based feature-selection framework was applied. For the nested models M1, M2, and HALO, feature selection was embedded within the training procedure rather than performed as a fixed preprocessing step: within each outer split, features were selected using only the corresponding inner-training folds during hyperparameter tuning, and feature selection was then rerun on the full outer-training set before final model refitting. As a result, the exact selected feature set could vary across folds and across model variants, reflecting differences in candidate feature space, grouping scheme, and training data composition. M3 used a fixed reduced feature set obtained upstream outside of CV folds. This feature selection was performed once on a drug-pair-held-out training split prior to model fitting, and the resulting feature subset was reused across all evaluation folds. Consequently, unlike M1, M2, and HALO, M3 evaluates a consistent but globally selected feature representation that introduces information leakage from outside the evaluation folds.

The strain-aware models (M2 and M1) used candidate features derived from both Chemical Checker and strain-space representations, whereas HALO, M3 and M4 used Chemical Checker features only.

For comparison, a lenient baseline (M4) was implemented using a standard stratified train/test split and standard stratified cross-validation without drug-pair grouping or nested outer-fold evaluation. Feature selection was performed once on the training subset only, and the reduced training representation was then used for standard hyperparameter tuning.

To assess whether the observed performance patterns were specific to this primary formulation, we additionally tested alternative model families and prediction tasks, including neural-network baselines, multi-class classification, regression on continuous interaction scores, and alternate feature encodings. We evaluated a feedforward neural-network baseline under identical CV, which underperformed LightGBM (accuracy 0.62 vs. 0.70; ROC-AUC 0.75 vs. 0.78; S3 Table), consistent with tree-based methods often outperforming deep architectures on small-to-moderate tabular datasets. We note that this baseline is not a graph neural network specifically; GNNs are typically most advantageous when learning directly from molecular graph structure, whereas our features are pre-computed Chemical Checker similarity embeddings rather than raw structural graphs, reducing the representational advantage GNNs would otherwise offer. Combined with our modest sample size (2,449 labeled interactions) and the interpretability benefits of gain-based feature importance for our mechanistic analyses (Section 2.4), these considerations motivated LightGBM as the primary model. A direct comparison against graph-based architectures on this dataset remains an important direction for future work. All of these exploratory analyses are reported in the S1 File and S3 Table.

### 3.5 Evaluation metrics and feature importance

Performance was assessed using accuracy, F1-macro, F1-weighted, ROC–AUC, and PR–AUC, with synergy treated as the positive class throughout. Confusion matrices were summarized over held-out predictions. Because the binary test sets were approximately balanced between synergy and antagonism, the random-chance baseline for PR–AUC was approximately 0.50. For the external INDIGO evaluation, the same classification metrics were computed, and ROC- and precision–recall curves were additionally generated from predicted synergy probabilities.

Feature importance for HALO was quantified using LightGBM gain-based importance, which measures each feature’s contribution to loss reduction across tree splits. For the nested models, feature importances were computed separately within each outer-fold final model and then aggregated across folds to obtain mean importance estimates. These importances were subsequently normalized and summarized across Chemical Checker (CC) sub-levels and higher-level CC domains. For M2, the contribution of strain-derived features was evaluated by grouping aggregated feature importances into CC-derived versus strain-space-derived features and comparing their normalized gain contributions (Fig 4D). To validate the robustness of the gain-based importance rankings, SHAP values [45] were computed for the final model using the shap Python package; the Spearman correlation between SHAP and gain-based rankings was calculated across all selected features.

### 3.6 Prediction of novel and external pairs

For final deployment-style analyses, HALO was retrained on the complete labeled dataset (2,449 drug-pair-strain binary combinations) using the best LightGBM hyperparameter setting obtained from nested cross-validation. Feature selection was performed once on the full labeled dataset using the same LightGBM-based feature importance procedure applied during model development, and the resulting subset of feature subset was used for all downstream predictions. The final trained model was then used to score all unlabeled drug–drug pairs generated from the antibacterial compound set, after excluding pairs already present in the labeled dataset. Predictions were aggregated by drug pair and ranked by predicted class probability to identify high confidence novel synergistic and antagonistic combinations.

External validation used the INDIGO dataset of Chandrasekaran et al. [19], which reports quantitative Loewe α interaction scores for antibiotic pairs in E. coli MG1655. After mapping compounds to InChIKeys, excluding non-antibacterials, binarizing labels, and retaining only pairs with CC coverage, 70 pairs remained. Labels were binarized as α < –0.5 for synergy and α > 1 for antagonism. These thresholds were taken directly from Chandrasekaran et al. [19], who reported that they provide a robust dose-integrated interaction measure and approximately correspond to strong Bliss-like deviations from additivity. Accordingly, we did not numerically convert Loewe α values into Bliss ε values; instead, we treated the external task as cross-assay validation at the level of binary interaction class. CC-based element-wise similarity features for the external set were generated with the same pipeline. To ensure strict external evaluation, we subset these features to the exact final feature set obtained from feature selection performed once on the full internal training dataset alone. No feature selection was performed on the external dataset.

The final HALO model trained on all internal training rows using the best hyperparameters identified during the internal nested CV experiments, was applied to the 70 externally derived feature vectors and external performance was evaluated using the same classification framework as in the internal analyses.

### 3.7 Software and reproducibility

All analyses were performed in Python 3.12.3 on a Linux-based high-performance server environment. Model training used LightGBM 4.6.0 and scikit-learn 1.7.2, pandas 2.3.3, numpy 2.3.5. and matplotlib 3.10.3. CC signatures were retrieved via the Chemical Checker API. Random seeds were fixed for all steps. Custom scripts and data are publicly available at https://github.com/Hannayousefabadi/HALO.

Additional methodological details, including alternative feature encodings, exploratory model variants, and neural-network baselines, are provided in the S1 File.

### 3.8 Computational cost and scalability

All runtime measurements were performed on a Linux server equipped with 64 logical cores (32 physical) and 270 GB RAM, running Python 3.9.23. The nested cross-validation workflow was profiled across five outer folds. Feature selection — using LightGBM-based iterative reduction within each outer training set — averaged 30.5 seconds per fold (total 152.5 s), while model fitting averaged 3.4 seconds per fold (total 16.8 s). Inference on the held-out test fold was negligible (0.1 seconds per fold), and a full external validation set (76 ACDB interactions) was scored in 7 milliseconds. Retraining the final model on the complete dataset required 36.8 seconds for feature selection and 4.0 seconds for model fitting. These timings demonstrate that while the leakage aware feature selection procedure is computationally nontrivial, it remains practical on standard academic hardware; once trained, HALO’s inference is highly efficient, enabling rapid screening of large combinatorial drug libraries.

## 4. Discussion

In this study, we propose a robust framework for machine-learning based modeling and prediction of antibacterial synergy. We first curated a large dataset of antibacterial drug–pair–strain interactions and leveraged the Chemical Checker drug features for model training. Under strict pair-held-out evaluation with nested cross-validation, HALO achieved stable and balanced performance on unseen drug combinations, demonstrating that multi-level bioactivity signatures alone provide a strong and interpretable basis for antibacterial interaction prediction.

Across evaluation schemes, we found that cross-validation strictness has a major impact on performance. The strain-aware M1 model performs poorly when required to generalize simultaneously to unseen drug pairs and unseen strains. With sparse strain coverage and limited replication of pair–strain combinations (only 10 strains, unevenly represented; Fig 1B), strain embeddings added little measurable signal beyond CC bioactivity features in our comparisons (M2 vs. HALO). We interpret this cautiously: this could reflect a genuine ceiling on the strain-level signal captured by our current embedding approach, or it could reflect insufficient statistical power to detect a real but sparse strain-specific effect given the limited strain diversity and replication available. Distinguishing between these possibilities will require training on datasets with substantially broader strain coverage. These findings clarify the practical scope of our approach. HALO is most suitable for prioritizing new drug combinations in well-characterized laboratory or clinical strains—an evaluation setting that aligns with many experimental screening workflows.

Beyond evaluation-protocol effects, several data- and design-level factors likely constrain HALO’s current predictive ability. First, as noted above, our training data span 97 antibacterial compounds and 10 bacterial strains — one of the most diverse curated bioactivity-based interaction sets currently available, though still modest relative to the full clinical antibiotic space. This represents a substantial expansion over prior antibacterial-specific ML datasets (see Introduction). HALO’s predictions should therefore be interpreted as most reliable within the chemical and taxonomic space represented in training, with possibly reduced confidence for structurally novel antibiotic classes or underrepresented species/strains. Second, label quality is itself a limiting factor: consistent with our rationale for excluding near-additive interactions (Section 3.2), most Bliss measurements cluster close to ε ≈ 0, where experimental noise is highest and true synergy or antagonism is hardest to distinguish, and much of the underlying data are dose-aggregated rather than dose-resolved — our predictor therefore cannot capture concentration-dependent shifts in interaction type, which are well documented for several drug classes. Third, our evaluation, while strict at the level of individual drug pairs, does not enforce structural or mechanism-of-action disjointness between training and test sets; held-out pairs may still share close scaffolds or mechanisms with pairs seen during training, which could inflate apparent generalization for structurally similar chemistries. Addressing these limitations will require larger, dose-resolved, and more standardized multi-strain datasets; richer strain-level features such as genomic or metabolic profiles; explicit scaffold- or mechanism-based splitting to more rigorously test extrapolation; and hybrid models that integrate CC bioactivity embeddings with mechanistic representations to improve both accuracy and interpretability.

In summary, HALO’s contributions are at multiple fronts: (1) To our knowledge, this is the first systematic application of CC-style, multi-level bioactivity embeddings to antibacterial pair-strain interactions; (2) We use strict evaluation by drug-pair-held-out nested cross-validation, explicitly quantifying — rather than assuming — the performance inflation introduced by more permissive schemes (Section 2.2); and (3) Our fold-internal feature selection avoids the leakage we demonstrate occurs when preprocessing is performed outside the cross-validation loop (M3 comparison, Section 2.2); (4) We built strain-aware model variants (M1, M2) that directly test — rather than assume — whether strain-level features improve prediction under matched evaluation conditions; and (5) Beyond these representational and evaluation differences, HALO’s curated dataset also substantially expands the scale of antibacterial interaction data available for model development and benchmarking: 2,449 labeled interactions across 97 compounds and 10 strains, compared to previous studies all using single-strains (Table 1).

Overall, the generalizability of HALO across independent experimental platforms underscores the robustness of the underlying CC representation, as well as the more robust cross-validation scheme used in this study during training compared to prior models. Overall, this study establishes a reliable baseline for antibacterial synergy prediction and a framework for benchmarking future models under realistic evaluation conditions. As larger and more diverse datasets emerge, this foundation will support increasingly accurate and mechanistically grounded computational discovery of antibacterial drug combinations.

### Declaration of generative AI and AI-assisted technologies in the manuscript preparation process

Large language models (ChatGPT 5.1 and Opus 4.5) were used to help edit and polish portions of the manuscript text. The authors revised and approved all content and are responsible for the scientific accuracy of the work.

## Supporting information

S1 Fig Sensitivity analysis of HALO’s performance to the Bliss neutrality cutoff used to define the synergy/antagonism threshold. (PNG)

S2 Fig Similarity features distributions in novel pairs and training pairs. (PNG)

S1 Table Comparison of HALO’s performance against standard stratified cross-validation (M4) and a partially drug-pair-held-out scheme with global feature selection (M3), showing inflated performance under both alternative evaluation schemes. (XLSX)

S2 Table Comparison of features selected by M3’s global feature-selection procedure versus HALO’s five independently selected fold-internal feature sets, illustrating feature-level evidence of selection leakage in M3. (XLSX)

S3 Table Comparison of binary classification against alternative task formulations (three-class classification and regression on continuous Bliss ε), and comparison of LightGBM against a feedforward neural-network baseline under identical cross-validation. (XLSX)

S4 Table Full ranked lists of predicted antagonistic drug pairs generated by the final HALO model for combinations absent from the training set. (XLSX)

S5 Table Full ranked lists of predicted synergistic drug pairs generated by the final HALO model for combinations absent from the training set. (XLSX)

S6 Table Mechanistic classification of the 57 conventional antibacterial compounds into 25 mechanistic categories (e.g., beta-lactams, macrolides, aminoglycosides, fluoroquinolones). (XLSX)

S1 File Additional methodological details, including alternative feature encodings, exploratory model variants, and neural-network baselines. (PDF)
