# Supplementary material for: Bioactivity-driven prediction of antibacterial synergy using machine learning models
Source: PLoS Comput Biol. 2026 Oct 5;22(10):e1014849. doi: 10.1371/journal.pcbi.1014849 (PMC13649120; doi:10.1371/journal.pcbi.1014849)
Supplement: Supplementary file 2 [file pcbi.1014849.s011.docx]

Response to Reviewers

Manuscript: Bioactivity-Driven Prediction of Antibacterial Synergy Using Machine Learning Models

Journal: PLOS Computational Biology

Dear Dr. Singh and Dr. Faeder,

We thank the editors and the three reviewers for their careful assessment of our

manuscript and for the constructive feedback. We have revised the manuscript

substantially in response to these comments. Below we address each point

individually. Reviewer comments are shown in italics/quotes, followed by our

response and, where applicable, the location of the corresponding change in

the revised manuscript.

Per the editor's note on the Journal Requirements, we have not included

responses to formatting/technical items here; those have been handled

directly in the resubmission (manuscript file format, figure files, and the

Competing Interest statement).

================================================================

REVIEWER 1

================================================================

Comment 1: The study introduces HALO and applies Chemical Checker-derived embeddings to antibacterial synergy prediction, the methodological innovation appears incremental relative to existing machine-learning frameworks such as INDIGO, CCSynergy, and graph-learning approaches. The authors should more clearly articulate the specific methodological advances and distinguish HALO from previously published bioactivity-based prediction models.

Response:

We thank the reviewer for this comment and have substantially revised the Introduction to more sharply articulate HALO's contribution relative to existing frameworks. We now explicitly distinguish HALO from INDIGO (which relies on *E. coli*-specific chemogenomic profiles with limited cross-species transfer), from structure-based and graph-learning approaches (which use molecular fingerprints without multi-level bioactivity context and are evaluated under permissive cross-validation schemes that allow substantial train/test overlap), and from CC-based frameworks in the cancer-synergy domain such as CCSynergy (which addresses a different task and evaluation design). We articulate four specific contributions: (1) the first systematic application of Chemical Checker-style, multi-level bioactivity embeddings to antibacterial pair-strain interactions; (2) evaluation exclusively under strict drug-pair-held-out (and, for one model variant, drug-pair-strain-held-out) nested cross-validation that explicitly quantifies rather than assumes the performance inflation introduced by more permissive schemes (Section 2.2); (3) fold-internal feature selection that avoids the leakage we directly demonstrate occurs when preprocessing is performed outside the cross-validation loop; and (4) systematic strain-aware model variants that directly test, rather than assume, whether strain-level features improve prediction under matched evaluation conditions. (See revised Introduction.)

Comment 2: The final dataset contains 3,160 interactions across only 10 bacterial strains, with many drug pairs observed in a single strain. This may substantially limit the ability to learn strain-dependent interaction patterns and raises concerns regarding the biological generalizability of the model. Authors hsould comment on this.

Response:

We agree that this is an important limitation and have expanded our discussion on this. In the Discussion, we explicitly note that our training data span only 97 compounds and 10 bacterial strains — modest relative to the full space of clinically relevant antibacterials and pathogens — and that HALO's predictions should be interpreted as most reliable within this represented chemical and taxonomic space, with reduced confidence for underrepresented species/strains or structurally novel antibiotic classes.

We also note that, relative to prior antibacterial-specific ML frameworks, HALO's dataset represents a substantial expansion in both chemical and strain diversity: INDIGO was trained on 105–171 pairwise interactions among 15–19 compounds in a single organism; CoSynE on 156 pairs among 18 compounds; and the graph-learning model of Lv et al. on 91 pairs among 14 antibiotics — all single-strain. We provide a structured qualitative comparison of feature representation, prediction target, dataset scale, and evaluation design across these methods in the manuscript (Table 1) and below. HALO's 2,449 labeled interactions span 97 compounds across 10 strains, representing the largest and most strain-diverse dataset among directly comparable antibacterial synergy prediction frameworks to date, even as we acknowledge this remains modest relative to the full space of clinically relevant antibiotics and pathogens.

# Table 1. Comparison of HALO with prior antibacterial-specific synergy prediction frameworks.

Feature representation, prediction target, dataset scale, and evaluation design differ substantially across methods, limiting the feasibility of a strict same-dataset, same-protocol benchmark (see Discussion). Reported metrics are each method's own published headline result and are not directly comparable across rows due to differing prediction tasks (continuous score, 3-class, or binary) and evaluation schemes.

Comment 3: The exclusion of all interactions with |ε| ≤ 0.1 removes potentially informative near-additive interactions and converts a continuous biological phenomenon into a binary classification problem. The authors should justify why regression or multiclass approaches were not prioritized and discuss the information loss associated with binarization.

Response:

We have added a direct empirical comparison (Results, Section 2.2; Supplementary Table S3) evaluating binary classification against both three-class classification (synergy/additive/antagonism) and regression on continuous Bliss ε, using the same feature set and CV scheme. Three-class classification underperformed substantially (accuracy 0.52, F1-macro 0.45 vs. 0.70/0.70 for HALO), with the disproportionate drop in F1-macro indicating the additive class is the primary source of difficulty. Regression performed comparably poorly (R² = 0.25, MAE = 0.17); notably, the regression model's average error (0.17) exceeds the ±0.1 margin used to define the additive band, meaning even continuous predictions could not reliably resolve which side of the boundary a given interaction falls on. We also now explicitly report that the exclusion removed 711 of 3,160 pair-strain instances (22.5%), and justify this threshold with reference to established synergy-scoring guidance and prior antibiotic-interaction literature, alongside a clinical-relevance argument for why the binary framing on the two actionable extremes (synergy to exploit, antagonism to avoid) is a deliberate choice rather than a convenience filter.

Comment 4: The independent validation ultimately includes only 70 antibacterial pairs, which may be insufficient to robustly establish model generalizability across experimental platforms and scoring methodologies. A suitable justification is required for this.

Response:

We agree that a single external validation set of limited size is insufficient to robustly establish generalizability, and have substantially expanded this analysis in two ways.

First, we identified that 36 of the original 70 INDIGO pairs (51.4%) shared a drug pair already present in our training data; we removed these and re-evaluated HALO on the remaining 34 genuinely novel pairs. Performance did not degrade on this stricter subset — HALO achieved a ROC-AUC of 0.90 (up from 0.84 on the original unfiltered set), indicating that HALO's external performance is not driven by pair-level memorization (Figure 5).

Second, we added an entirely independent external validation using a subset of the Antibiotic Combination Database (ACDB) that was not used during training. While Bliss-scored ACDB interactions were incorporated into our primary training data, a separate subset scored via the FICI or Loewe methods was held out and never used for training. After the same drug-pair overlap removal applied to INDIGO and restricting to clear synergy/antagonism outcomes, this yielded 76 pair-strain instances across 54 antibacterials. HALO achieved a ROC-AUC of 0.71 on this set (Figure 6).

Together, these two validations span two independent data sources, two interaction-scoring methodologies distinct from our Bliss-based training data (Loewe α and FICI), and markedly different class distributions (INDIGO: 18% synergy; ACDB: 87% synergy) — with all evaluated pairs confirmed absent from training. We believe this substantially strengthens the evidence for HALO's generalizability across assay types and scoring conventions, while acknowledging that further validation on additional independent datasets, as also raised by Reviewers 2 and 3, remains valuable future work.

Comment 5: Although the manuscript discusses prior models, direct benchmarking against contemporary approaches using the same curated dataset and identical pair-held-out validation schemes is lacking.

Response:

We appreciate this comment and want to be transparent about why a strict same-dataset, same-protocol benchmark against INDIGO, CoSynE, and related methods is not statistically or methodologically feasible, despite genuine effort to pursue it. These frameworks differ from HALO not only in feature representation (chemogenomic fitness profiles, structural fingerprints, or molecular graphs, versus multi-level bioactivity embeddings) but also, for INDIGO and CoSynE specifically, prediction targets are different as well (continuous interaction scores rather than binary classification). These frameworks' original datasets (91–171 pairs among 14–19 compounds) are too small to support the nested pair-held-out cross-validation with independent hyperparameter tuning that we advocate for as a methodological standard (Section 2.2) — a full nested design would leave too few samples per inner fold for reliable tuning. We provide a structured qualitative comparison of feature representation, prediction target, dataset scale, and evaluation design across these methods in the manuscript (Table 1) and below.

# Table 1. Comparison of HALO with prior antibacterial-specific synergy prediction frameworks.

Feature representation, prediction target, dataset scale, and evaluation design differ substantially across methods, limiting the feasibility of a strict same-dataset, same-protocol benchmark (see Discussion). Reported metrics are each method's own published headline result and are not directly comparable across rows due to differing prediction tasks (continuous score, 3-class, or binary) and evaluation schemes.

As the closest feasible same-dataset, same-protocol comparison, we have now retrained HALO's own pipeline with a Random Forests classifier — matching the algorithm family underlying CoSynE and INDIGO — while keeping HALO's feature selection procedure, dataset, and nested cross-validation schemes. For each of these benchmarking models the feature set is a subset of Chemical Checker database that best matches the original platform (A4 structural key features for CoSynE; D3 chemical genetics features for INDIGO). Both of these narrow-feature RF variants underperformed HALO's full multi-level model (Figure 7 of manuscript): The CoSynE-style achieved accuracy = 0.66 and ROC-AUC = 0.72, and the INDIGO-style achieved accuracy = 0.65 and ROC-AUC = 0.72, compared to HALO's accuracy = 0.70 and ROC-AUC = 0.78. Halo outperformed both models in accuracy and roc-auc. We have added this result to section 2.5 external validation and algorithm benchmarking.

**Figure 7.** **Benchmarking against CoSynE- and INDIGO-representative baselines.**

HALO's full multi-level Chemical Checker representation was compared against two narrow-feature Random Forest baselines under identical nested pair-held-out cross-validation: one restricted to CC sublevel A4 (structural keys), resembling CoSynE's structural fingerprint representation, and one restricted to CC sublevel D3 (chemical genetics), resembling INDIGO's chemogenomic representation. (A) Accuracy and (B) ROC-AUC, mean ± SD across five outer folds. HALO outperformed both narrow-feature baselines on both metrics.

Regarding Lv et al. specifically: their method is not a supervised classifier trained on labeled feature vectors, but a semi-supervised label-propagation algorithm operating directly over a protein-protein interaction network topology. This is a fundamentally different input modality (network structure rather than compound-level bioactivity features) and learning paradigm from HALO, CoSynE, and INDIGO alike, making a like-for-like retraining within HALO's pipeline architecturally inapplicable rather than merely impractical. We report its published metrics and dataset characteristics qualitatively in Table 1 of manuscript for reference.

Comment 6: The rationale for selecting LightGBM as the primary model should be explained in greater detail, particularly relative to graph neural networks and deep learning approaches.

Response:

We have expanded Section 3.4 to include a direct comparison against a feedforward neural-network baseline evaluated under identical cross-validation, which underperformed LightGBM (accuracy 0.62 vs. 0.70; ROC-AUC 0.75 vs. 0.78; Supplementary Table S3). We also now explicitly note that this baseline is not a graph neural network specifically, and discuss why GNNs are less likely to offer a representational advantage here: our features are pre-computed Chemical Checker similarity embeddings rather than raw molecular graph structure, which reduces the benefit GNNs typically provide. Combined with our modest sample size (2,449 labeled interactions) and the interpretability benefits of gain-based feature importance for our mechanistic analyses, these considerations motivated LightGBM as the primary model. We note that a direct comparison against graph-based architectures remains an important direction for future work.

Comment 7: It is unclear whether class imbalance handling techniques were considered during model training, particularly for external validation where synergistic interactions constitute only 19% of samples.

Response:

We have clarified this in two places. In Results (Section 2.1), we now state that our internal binary training set was near-balanced (1,222 synergy vs. 1,227 antagonism, ~50/50), so no explicit class-reweighting or resampling was applied during training. In Results (Section 2.5), we clarify that the external INDIGO-derived validation set was class-imbalanced (19% synergy) as a natural consequence of that independent dataset's own interaction distribution, not something we controlled for, and that we report PR-AUC/average precision alongside ROC-AUC there specifically because these metrics are more informative than accuracy under class imbalance.

Comment 8: Authors can also improve the overall introduction and discussion by incorporating recent literaure. For the same i have given few: PMID: 34365920, PMID: 34916893.

Response:

We thank the reviewers for these suggestions and reviewed each reference. we have cited it in the Introduction as an example of the broader landscape of ML-based drug-relationship prediction.

Comment 9: More information regarding the distribution of antibacterial classes in training and validation datasets would help assess potential chemical-space biases.

Response:

As described now in the manuscript section 2.1, out of the 97 compounds in our dataset, 57 belong to conventional antibacterial classes spanning 25 mechanistic categories (beta-lactams, macrolides, aminoglycosides, fluoroquinolones, and others; Supplementary Table S6), while 40 are non-traditional antibacterial agents — compounds originally developed for other indications (e.g., NSAIDs, antipsychotics, antineoplastics) but with independently documented direct antibacterial activity, consistent with the growing literature on repurposed drugs as antimicrobials. Beta-lactamase inhibitors and other potentiator-only compounds without direct antibacterial activity were excluded from curation. Because HALO's evaluation holds out drug pairs rather than individual compounds, both categories are represented in training and test partitions across all outer folds, limiting the potential for class-level chemical-space bias.

Comment 10: Exact p-values should be reported whenever statistical comparisons are performed.

Response:

We have added exact statistics for all model comparisons in Results (Section 2.2) and Supplementary Table S1: HALO vs. M3 (paired t-test, p ≈ 0.002 across all metrics, demonstrating the leakage effect of global feature selection), HALO vs. M4 (one-sample t-test against HALO's fold distribution, p = 0.004–0.011, demonstrating performance inflation under standard non-grouped CV), and HALO vs. M2 (paired t-test, p = 0.26–0.81, showing no statistically significant contribution from strain-space features under this evaluation scheme).

================================================================

REVIEWER 2

================================================================

Reviewer 2's comments were not individually numbered in the original review; we have

numbered them below in the order presented for clarity of response.

Comment 1: The novelty of the proposed HALO framework appears to stem primarily from the evaluation strategy rather than the predictive model itself. The authors should more clearly distinguish the methodological novelty from existing Chemical Checker-based approaches and provide a direct comparison with state-of-the-art models under the same stringent validation framework.

Response:

We have substantially revised the Introduction to articulate HALO's contributions more precisely, and we now explicitly distinguish between (i) the representational innovation, (ii) the evaluation-rigor innovation, and (iii) the dataset-scale contribution. We emphasize that HALO is the first framework to systematically apply Chemical Checker (CC)-style, multi-level bioactivity embeddings to antibacterial synergy prediction. We now explicitly distinguish HALO's representation (the first systematic application of Chemical Checker-style, multi-level bioactivity embeddings to antibacterial pair-strain interactions) from INDIGO's chemogenomic profiles and CoSynE's structural fingerprints, in addition to our evaluation-rigor contributions (strict pair-held-out nested cross-validation, and fold-internal feature selection avoiding the leakage we directly demonstrate in Section 2.2).

We have also added a structured comparison table to the manuscript (Table 1) and also below, contrasting HALO with INDIGO, CoSynE, and the network-based model of Lv et al. across feature representation, prediction target, dataset scale, and evaluation design.

# Table 1. Comparison of HALO with prior antibacterial-specific synergy prediction frameworks.

Feature representation, prediction target, dataset scale, and evaluation design differ substantially across methods, limiting the feasibility of a strict same-dataset, same-protocol benchmark (see Discussion). Reported metrics are each method's own published headline result and are not directly comparable across rows due to differing prediction tasks (continuous score, 3-class, or binary) and evaluation schemes.

As the closest feasible same-dataset, same-protocol comparison, we have now retrained HALO's own pipeline with a Random Forests classifier — matching the algorithm family underlying CoSynE and INDIGO — while keeping HALO's feature selection procedure, dataset, and nested cross-validation schemes. For each of these benchmarking models the feature set is a subset of Chemical Checker database that best matches the original platform (A4 structural key features for CoSynE; D3 chemical genetics features for INDIGO). Both of these narrow-feature RF variants underperformed HALO's full multi-level model (Figure 7 of manuscript): The CoSynE-style achieved accuracy = 0.66 and ROC-AUC = 0.72, and the INDIGO-style achieved accuracy = 0.65 and ROC-AUC = 0.72, compared to HALO's accuracy = 0.70 and ROC-AUC = 0.78. Halo outperformed both models in accuracy and roc-auc. We have added this result to section 2.5 external validation and algorithm benchmarking.

**Figure 7.** **Benchmarking against CoSynE- and INDIGO-representative baselines.**

HALO's full multi-level Chemical Checker representation was compared against two narrow-feature Random Forest baselines under identical nested pair-held-out cross-validation: one restricted to CC sublevel A4 (structural keys), resembling CoSynE's structural fingerprint representation, and one restricted to CC sublevel D3 (chemical genetics), resembling INDIGO's chemogenomic representation. (A) Accuracy and (B) ROC-AUC, mean ± SD across five outer folds. HALO outperformed both narrow-feature baselines on both metrics.

Regarding Lv et al. specifically: their method is not a supervised classifier trained on labeled feature vectors, but a semi-supervised label-propagation algorithm operating directly over a protein-protein interaction network topology. This is a fundamentally different input modality (network structure rather than compound-level bioactivity features) and learning paradigm from HALO, CoSynE, and INDIGO alike, making a like-for-like retraining within HALO's pipeline architecturally inapplicable rather than merely impractical. We report its published metrics and dataset characteristics qualitatively in Table 1 of manuscript for reference.

Comment 2: The manuscript reports performance using a single curated dataset containing only 97 antibacterial compounds and 10 strains. The authors should discuss the limitations imposed by the relatively small chemical and biological diversity of the dataset and provide confidence intervals or statistical significance testing for the reported ROC-AUC values to better assess model robustness.
Response:

We have expanded our discussion of dataset diversity limits in the Discussion, noting that our training data span only 97 compounds and 10 bacterial strains and that HALO's predictions should be interpreted as most reliable within this represented space. We have also added exact statistics for our model comparisons, including confidence-interval-equivalent significance testing for ROC-AUC differences (paired and one-sample t-tests; Results Section 2.2, Supplementary Table S1).

We should also emphasize our dataset's scale relative to existing resources. While the Antibiotic Combination Database (ACDB) contains a larger raw compilation (6,175 combinations across 304 compounds and 460 strains), it is manually aggregated from hundreds of independent literature studies, each using its own strain, protocol, and interaction-scoring convention (predominantly checkerboard/FICI), without a single harmonized quantitative interaction metric across entries. HALO's 2,449 interactions, by contrast, are harmonized under a single quantitative framework (Bliss independence) with a reproducible cross-study replicate-resolution procedure and a consistent CC-derived feature representation across all compounds (Section 2.1). To our knowledge, this makes HALO's training set the largest *harmonized, single-methodology* dataset assembled for antibacterial synergy prediction to date — the property that specifically enables the rigorous nested pair-held-out evaluation protocol we advocate for (Section 2.2), which heterogeneous compilations like ACDB cannot directly support without substantial reprocessing (as we in fact performed to construct our ACDB-derived external validation set; Section 2.5).

Comment 3: The manuscript excludes all interactions with |ε| ≤ 0.1 before classification, thereby removing a substantial proportion of biologically relevant near-additive interactions. The authors should quantify how many samples were excluded and evaluate whether the model remains effective under a more realistic multi-class or continuous prediction setting rather than only classifying extreme synergy and antagonism.

Response:

We have added a direct empirical comparison (Results, Section 2.2; Supplementary Table S3) evaluating binary classification against both three-class classification (synergy/additive/antagonism) and regression on continuous Bliss ε, using the same feature set and CV scheme. Three-class classification underperformed substantially (accuracy 0.52, F1-macro 0.45 vs. 0.70/0.70 for HALO), with the disproportionate drop in F1-macro indicating the additive class is the primary source of difficulty. Regression performed comparably poorly (R² = 0.25, MAE = 0.17); notably, the regression model's average error (0.17) exceeds the ±0.1 margin used to define the additive band, meaning even continuous predictions could not reliably resolve which side of the boundary a given interaction falls on. We also now explicitly report that the exclusion removed 711 of 3,160 pair-strain instances (22.5%), and justify this threshold with reference to established synergy-scoring guidance and prior antibiotic-interaction literature, alongside a clinical-relevance argument for why the binary framing on the two actionable extremes (synergy to exploit, antagonism to avoid) is a deliberate choice rather than a convenience filter.

Comment 4: The authors claim that global feature selection in M3 introduces information leakage, but the extent of this leakage is not rigorously quantified. A formal comparison showing the number of selected features, overlap across folds, and statistical differences between M3 and HALO would strengthen the argument regarding the importance of fold-internal feature selection.

Response:

We have added a direct, feature-level quantification of this leakage (Results, Section 2.2; Supplementary Table S2). M3's globally selected feature set was identical across all five outer folds (Jaccard overlap = 1.000 for every fold pair), confirming that its single global selection step is reused without adaptation to each fold's training data. HALO's fold-internal selection yielded a comparable mean feature count (1,609 ± 7 vs. M3's fixed 1,600) but substantially lower fold-to-fold consistency (mean Jaccard = 0.296 ± 0.009), reflecting genuine, data-dependent reselection. Critically, 226 features (14.1%) present in M3's global set were never independently selected in any of HALO's five folds, despite being drawn from the same feature pool — these features are accessible to M3 only because its selection procedure had visibility into the full dataset, including instances later held out for testing. Combined with our existing statistical comparison of predictive performance (paired t-test, p ≈ 0.002 across all metrics; Section 2.2), this provides both a mechanistic and quantitative account of the leakage effect.

Comment 5: The manuscript uses gain-based LightGBM feature importance as the primary interpretability method. The authors should consider complementing this analysis with SHAP values or permutation-based importance because gain importance can be biased toward features with higher variability and may not accurately reflect biological relevance. Refer to this article: PMID-39657879 to improve the methodology, result, and discussion sections which will be more beneficial for the readership of the article and journal.

Response:

We have cited it in the Introduction as an example of the broader landscape of ML-based drug-relationship prediction (reference 17). To address the substantive methodological point, we performed a SHAP analysis following the established framework of Lundberg & Lee (NeurIPS 2017). Specifically, we computed SHAP values for the final HALO model and compared the resulting feature rankings with the LightGBM gain-based importances. The SHAP-based ranking was positively correlated with the gain-based ordering (Spearman ρ = 0.66), confirming a non-random association. Importantly, while the fine-grained ordering of individual features differed both approaches consistently identified the same high-level Chemical Checker sub-levels.

Comment 6: The authors conclude that strain embeddings contribute little predictive information. However, this conclusion may be confounded by sparse strain coverage and limited overlap of drug pairs across strains. Additional analyses examining strain-specific performance or incorporating genomic and phenotypic strain descriptors would be necessary before dismissing the value of strain-level information.

Response:

We agree this conclusion required more careful framing and have revised the Discussion accordingly: strain embeddings added little measurable signal beyond CC bioactivity features in our comparisons, but we now explicitly note this could reflect either a genuine ceiling on the strain-level signal captured by our current embedding approach, or insufficient statistical power given the limited strain diversity and replication in our dataset (only 10 strains, unevenly represented). Distinguishing between these possibilities will require training on datasets with substantially broader strain coverage; we have not yet incorporated genomic or phenotypic strain descriptors, which we note as a valuable future direction.

Comment 7: The manuscript reports successful external validation on the INDIGO dataset, yet only 70 antibacterial pairs remained after filtering. The authors should discuss the limited size of this validation cohort and evaluate whether any compounds or drug classes overlap with the training data, as such overlap could partially inflate external performance estimates.

Response:

We agree that a single external validation set of limited size is insufficient to robustly establish generalizability, and have substantially expanded this analysis in two ways.

First, we identified that 36 of the original 70 INDIGO pairs (51.4%) shared a drug pair already present in our training data; we removed these and re-evaluated HALO on the remaining 34 genuinely novel pairs. Performance did not degrade on this stricter subset — HALO achieved a ROC-AUC of 0.90 (up from 0.84 on the original unfiltered set), indicating that HALO's external performance is not driven by pair-level memorization (Figure 5).

Second, we added an entirely independent external validation using a subset of the Antibiotic Combination Database (ACDB) that was not used during training. While Bliss-scored ACDB interactions were incorporated into our primary training data, a separate subset scored via the FICI or Loewe methods was held out and never used for training. After the same drug-pair overlap removal applied to INDIGO and restricting to clear synergy/antagonism outcomes, this yielded 76 pair-strain instances across 54 antibacterials. HALO achieved a ROC-AUC of 0.71 on this set (Figure 6).

Together, these two validations span two independent data sources, two interaction-scoring methodologies distinct from our Bliss-based training data (Loewe α and FICI), and markedly different class distributions (INDIGO: 18% synergy; ACDB: 87% synergy) — with all evaluated pairs confirmed absent from training. We believe this substantially strengthens the evidence for HALO's generalizability across assay types and scoring conventions, while acknowledging that further validation on additional independent datasets, as also raised by Reviewers 2 and 3, remains valuable future work.

Comment 8: The manuscript presents several high-confidence novel synergistic predictions and extensive mechanistic interpretations. However, no experimental validation is provided. The authors should moderate their conclusions and clearly state that these combinations remain computational hypotheses until verified through in vitro or in vivo testing.

Response:

We agree and have revised our framing of novel predicted pairs throughout the Results. These are now explicitly described as mechanistically plausible computational hypotheses generated from bioactivity-based pattern recognition, not experimentally confirmed interactions, and we state that wet-lab validation (e.g., checkerboard assays) is required before any of these pairs can be considered candidates for further pharmacological development.

================================================================

REVIEWER 3

================================================================

Comment 1: The dataset used for model development is relatively limited in size and diversity (97 antibacterial compounds and 3,160 drug–pair–strain interactions). Although the authors acknowledge this limitation, a more detailed discussion on how the dataset size may affect model generalizability and robustness is warranted. Additional validation on larger or more diverse datasets would strengthen the conclusions.

Response:

We agree this is an important limitation and have expanded our discussion. In the Discussion, we explicitly note that our training data span only 97 compounds and 10 bacterial strains — modest relative to the full space of clinically relevant antibacterials and pathogens — and that HALO's predictions should be interpreted as most reliable within this represented chemical and taxonomic space, with reduced confidence for underrepresented species/strains or structurally novel antibiotic classes.

We also note that, relative to prior antibacterial-specific ML frameworks, HALO's dataset represents a substantial expansion in both chemical and strain diversity: INDIGO was trained on 105–171 pairwise interactions among 15–19 compounds in a single organism; CoSynE on 156 pairs among 18 compounds; and the graph-learning model of Lv et al. on 91 pairs among 14 antibiotics — all single-strain. HALO's 2,449 labeled interactions span 97 compounds across 10 strains, representing the largest and most strain-diverse dataset among directly comparable antibacterial synergy prediction frameworks to date, even as we acknowledge this remains modest relative to the full space of clinically relevant antibiotics and pathogens (Table 1 below – in response to comment 2).

Comment 2: The manuscript primarily compares different evaluation schemes rather than benchmarking HALO against recent state-of-the-art antibacterial synergy prediction models. A direct comparison using the same dataset and evaluation protocol would provide a clearer assessment of its relative performance.

Response:

We appreciate this comment and want to be transparent about why a strict same-dataset, same-protocol benchmark against INDIGO, CoSynE, and related methods is not statistically or methodologically feasible, despite genuine effort to pursue it. These frameworks differ from HALO not only in feature representation (chemogenomic fitness profiles, structural fingerprints, or molecular graphs, versus multi-level bioactivity embeddings) but, for INDIGO and CoSynE specifically, in prediction target as well (continuous interaction scores rather than binary classification). None of these frameworks' original implementations are publicly available, and their datasets (91–171 pairs among 14–19 compounds) are too small to support the nested pair-held-out cross-validation with independent hyperparameter tuning that we advocate for as a methodological standard (Section 2.2). We provide a structured qualitative comparison of feature representation, prediction target, dataset scale, and evaluation design across these methods in the manuscript (Table 1) and below.

# Table 1. Comparison of HALO with prior antibacterial-specific synergy prediction frameworks.

Feature representation, prediction target, dataset scale, and evaluation design differ substantially across methods, limiting the feasibility of a strict same-dataset, same-protocol benchmark (see Discussion). Reported metrics are each method's own published headline result and are not directly comparable across rows due to differing prediction tasks (continuous score, 3-class, or binary) and evaluation schemes.

As the closest feasible same-dataset, same-protocol comparison, we have now retrained HALO's own pipeline with a Random Forests classifier — matching the algorithm family underlying CoSynE and INDIGO — while keeping HALO's feature selection procedure, dataset, and nested cross-validation schemes. For each of these benchmarking models the feature set is a subset of Chemical Checker database that best matches the original platform (A4 structural key features for CoSynE; D3 chemical genetics features for INDIGO). Both of these narrow-feature RF variants underperformed HALO's full multi-level model (Figure 7 of manuscript): The CoSynE-style achieved accuracy = 0.66 and ROC-AUC = 0.72, and the INDIGO-style achieved accuracy = 0.65 and ROC-AUC = 0.72, compared to HALO's accuracy = 0.70 and ROC-AUC = 0.78. Halo outperformed both models in accuracy and roc-auc. We have added this result to section 2.5 external validation and algorithm benchmarking.

**Figure 7.** **Benchmarking against CoSynE- and INDIGO-representative baselines.**

HALO's full multi-level Chemical Checker representation was compared against two narrow-feature Random Forest baselines under identical nested pair-held-out cross-validation: one restricted to CC sublevel A4 (structural keys), resembling CoSynE's structural fingerprint representation, and one restricted to CC sublevel D3 (chemical genetics), resembling INDIGO's chemogenomic representation. (A) Accuracy and (B) ROC-AUC, mean ± SD across five outer folds. HALO outperformed both narrow-feature baselines on both metrics.

Regarding Lv et al. specifically: their method is not a supervised classifier trained on labeled feature vectors, but a semi-supervised label-propagation algorithm operating directly over a protein-protein interaction network topology. This is a fundamentally different input modality (network structure rather than compound-level bioactivity features) and learning paradigm from HALO, CoSynE, and INDIGO alike, making a like-for-like retraining within HALO's pipeline architecturally inapplicable rather than merely impractical. We report its published metrics and dataset characteristics qualitatively in Table 1 of manuscript for reference.

Comment 3: While the study emphasizes leakage-free evaluation, it remains unclear whether structurally or mechanistically similar drug pairs could still appear across training and test folds. The authors should discuss the potential influence of chemically related compounds on model performance and whether scaffold- or mechanism-based splitting strategies were considered.

Response:

We have added a discussion of this point. Our current outer-fold splits enforce held-out drug pairs (and, for one model variant, held-out strains), but do not explicitly enforce structural or mechanism-of-action disjointness; held-out pairs may therefore still share close scaffolds or mechanisms with pairs seen during training. We note this in the Discussion as a limitation and identify scaffold- or mechanism-based splitting as an important direction for future work to more rigorously test extrapolation to structurally or mechanistically novel drug pairs.

Comment 4: The external validation is performed on only 70 drug pairs from the INDIGO dataset. Although encouraging, this relatively small external benchmark limits the strength of the generalization claims. Validation on additional independent datasets, bacterial species, or experimental platforms would strengthen the MS.

Response:

We agree that a single external validation set of limited size is insufficient to robustly establish generalizability, and have substantially expanded this analysis in two ways.

First, we identified that 36 of the original 70 INDIGO pairs (51.4%) shared a drug pair already present in our training data; we removed these and re-evaluated HALO on the remaining 34 genuinely novel pairs. Performance did not degrade on this stricter subset — HALO achieved a ROC-AUC of 0.90 (up from 0.84 on the original unfiltered set), indicating that HALO's external performance is not driven by pair-level memorization (Figure 5).

Second, we added an entirely independent external validation using a subset of the Antibiotic Combination Database (ACDB) that was not used during training. While Bliss-scored ACDB interactions were incorporated into our primary training data, a separate subset scored via the FICI or Loewe methods was held out and never used for training. After the same drug-pair overlap removal applied to INDIGO and restricting to clear synergy/antagonism outcomes, this yielded 76 pair-strain instances across 54 antibacterials. HALO achieved a ROC-AUC of 0.71 on this set (Figure 6).

Together, these two validations span two independent data sources, two interaction-scoring methodologies distinct from our Bliss-based training data (Loewe α and FICI), and markedly different class distributions (INDIGO: 18% synergy; ACDB: 87% synergy) — with all evaluated pairs confirmed absent from training. We believe this substantially strengthens the evidence for HALO's generalizability across assay types and scoring conventions, while acknowledging that further validation on additional independent datasets, as also raised by Reviewers 2 and 3, remains valuable future work.

Comment 5: The model predicts binary synergy versus antagonism after excluding additive interactions (|ε| ≤ 0.1). Since most experimentally observed drug combinations are additive, excluding this large category may reduce the model's practical applicability. The authors should discuss this limitation and consider evaluating multi-class or continuous interaction prediction in greater detail.

Response:

We have added a direct empirical comparison (Results, Section 2.2; Supplementary Table S3) evaluating binary classification against both three-class classification (synergy/additive/antagonism) and regression on continuous Bliss ε, using the same feature set and CV scheme. Three-class classification underperformed substantially (accuracy 0.52, F1-macro 0.45 vs. 0.70/0.70 for HALO), with the disproportionate drop in F1-macro indicating the additive class is the primary source of difficulty. Regression performed comparably poorly (R² = 0.25, MAE = 0.17); notably, the regression model's average error (0.17) exceeds the ±0.1 margin used to define the additive band, meaning even continuous predictions could not reliably resolve which side of the boundary a given interaction falls on. We also now explicitly report that the exclusion removed 711 of 3,160 pair-strain instances (22.5%), and justify this threshold with reference to established synergy-scoring guidance and prior antibiotic-interaction literature, alongside a clinical-relevance argument for why the binary framing on the two actionable extremes (synergy to exploit, antagonism to avoid) is a deliberate choice rather than a convenience filter.

Comment 6: Experimental validation of at least a subset of the newly predicted synergistic combinations would substantially increase the biological significance and translational impact of the work.

Response:

We completely agree with the reviewer that experimental validation would substantially strengthen the translational impact of the study. However, the primary focus of this work is to establish a robust computational framework and rigorous evaluation protocol for antibacterial synergy prediction, rather than to perform prospective wetlab screening. To make this limitation explicit, we have clearly stated in the manuscript that all predicted pairs are *in silico* hypotheses requiring experimental followup. Specifically, in the final paragraph of **Section 2.6 (Novel drug-pair discoveries)**, we write: “*We also emphasize that these remain computational hypotheses… wetlab validation (e.g., checkerboard assays) is required before any of these pairs can be considered candidates for further pharmacological development.”*

Comment 7: The manuscript concludes that strain embeddings contribute minimally to prediction; however, this may largely reflect the limited strain coverage rather than the lack of biological relevance. The authors should discuss whether richer strain-specific features, such as genomic, transcriptomic, or metabolic profiles, could improve predictive performance.

Response:

We agree this conclusion required more careful framing and have revised the Discussion accordingly: strain embeddings added little measurable signal beyond CC bioactivity features in our comparisons, but we now explicitly note this could reflect either a genuine ceiling on the strain-level signal captured by our current embedding approach, or insufficient statistical power given the limited strain diversity and replication in our dataset (only 10 strains, unevenly represented). Distinguishing between these possibilities will require training on datasets with substantially broader strain coverage; we have not yet incorporated genomic or phenotypic strain descriptors, which we note as a valuable future direction.

Comment 8: Although LightGBM was selected as the final model, the manuscript provides only limited justification for this choice. A more comprehensive comparison with recent deep learning approaches (e.g., graph neural networks or transformer-based architectures) under identical validation settings would strengthen the methodological rationale.

Response:

We thank the reviewer for this comment. We agree that model justification is important and had in fact addressed this in the original submission, though we recognize it may not have been sufficiently prominent. In Section 3.4 (paragraph beginning "To assess whether the observed performance patterns were specific to this primary formulation..."), we report that a feedforward neural-network baseline evaluated under the identical nested cross-validation protocol underperformed LightGBM (accuracy 0.62 vs. 0.70; ROC-AUC 0.75 vs. 0.78; Supplementary Table S3). We also explain our rationale for not additionally benchmarking a graph neural network or transformer architecture: our input features are precomputed Chemical Checker similarity embeddings rather than raw molecular graph structures, which substantially reduces the representational advantage such architectures typically confer. Combined with our modest sample size (2,449 labeled interactions) and our reliance on LightGBM's gain-based feature importance for the mechanistic analyses in Section 2.4, these considerations motivated LightGBM as our primary model. We have revised the text to make this justification more visible to the reader.

Comment 9: While the proposed framework is computationally efficient compared to deep learning methods, the manuscript does not provide information regarding computational cost, runtime, or scalability.

Response:

We thank the reviewer for this important point and have now included a detailed computational cost analysis (section 3.7). All timing experiments were performed on a Linux server with 64 logical cores (32 physical), 270 GB RAM, using Python 3.9.23.

================================================================

CLOSING

================================================================

We believe these revisions have substantially strengthened the manuscript

and thank the reviewers again for their time and thoughtful comments.

Sincerely,

Mahya Mehrmohamadi

On behalf of all authors
