# Supplementary material for: Bioactivity-driven prediction of antibacterial synergy using machine learning models
Source: PLoS Comput Biol. 2026 Oct 5;22(10):e1014849. doi: 10.1371/journal.pcbi.1014849 (PMC13649120; doi:10.1371/journal.pcbi.1014849)
Supplement: Peer review history

# Peer review: Decision Letter 0

**DOI:** 10.1371/journal.pcbi.1014849.r001

**Authors:** James Faeder, Rahul Singh

3 Jul 2026

Bioactivity-Driven Prediction of Antibacterial Synergy Using Machine Learning Models

PLOS Computational Biology

Dear Dr. Mehrmohamadi,

Thank you for submitting your manuscript to PLOS Computational Biology. After careful consideration, we feel that it has merit but does not fully meet PLOS Computational Biology's publication criteria as it currently stands. Therefore, we invite you to submit a revised version of the manuscript that addresses the points raised during the review process.

Please submit your revised manuscript by Sep 01 2026 11:59PM. If you will need more time than this to complete your revisions, please reply to this message or contact the journal office at ploscompbiol@plos.org. When you're ready to submit your revision, log on to https://www.editorialmanager.com/pcompbiol/ and select the 'Submissions Needing Revision' folder to locate your manuscript file.

Please include the following items when submitting your revised manuscript:

* A letter that responds to each point raised by the editor and reviewer(s). You should upload this letter as a separate file labeled 'Response to Reviewers'. This file does not need to include responses to formatting updates and technical items listed in the 'Journal Requirements' section below.

* A marked-up copy of your manuscript that highlights changes made to the original version. You should upload this as a separate file labeled 'Revised Manuscript with Track Changes'.

* An unmarked version of your revised paper without tracked changes. You should upload this as a separate file labeled 'Manuscript'.

Kind regards,

Rahul Singh, Ph.D.

Academic Editor

PLOS Computational Biology

James Faeder

Section Editor

PLOS Computational Biology

**Additional Editor Comments (if provided):**

**Journal Requirements:**

If the reviewer comments include a recommendation to cite specific previously published works, please review and evaluate these publications to determine whether they are relevant and should be cited. There is no requirement to cite these works unless the editor has indicated otherwise.

1) We ask that a manuscript source file is provided at Revision. Please upload your manuscript file as a .doc, .docx, .rtf or .tex. If you are providing a .tex file, please upload it under the item type u2018LaTeX Source Fileu2019 and leave your .pdf version as the item type u2018Manuscriptu2019.

2) Please upload all main figures as separate Figure files in .tif or .eps format. For more information about how to convert and format your figure files please see our guidelines:

https://journals.plos.org/ploscompbiol/s/figures.

3)Please upload a copy of Figures FIG 1A-C, 2A-D, 3A-E, 4A-D, and 5A-D which you refer to in your text on pages 6, 9, 13, 15, and 17 . Or, if the figure is no longer to be included as part of the submission please remove all reference to it within the text.

4)We have amended your Competing Interest statement to comply with journal style. We kindly ask that you double check the statement and let us know if anything is incorrect.

**Reviewers' comments:**

Reviewer's Responses to Questions

**Comments to the Authors:**

**Please note here if the review is uploaded as an attachment.**

Reviewer #1: Authors have done good work but few things need to be addressed before final acceptance. Here are the few suggested changes:

1. The study introduces HALO and applies Chemical Checker-derived embeddings to antibacterial synergy prediction, the methodological innovation appears incremental relative to existing machine-learning frameworks such as INDIGO, CCSynergy, and graph-learning approaches. The authors should more clearly articulate the specific methodological advances and distinguish HALO from previously published bioactivity-based prediction models.

2. The final dataset contains 3,160 interactions across only 10 bacterial strains, with many drug pairs observed in a single strain. This may substantially limit the ability to learn strain-dependent interaction patterns and raises concerns regarding the biological generalizability of the model. Authors hsould comment on this.

3. The exclusion of all interactions with |ε| ≤ 0.1 removes potentially informative near-additive interactions and converts a continuous biological phenomenon into a binary classification problem. The authors should justify why regression or multiclass approaches were not prioritized and discuss the information loss associated with binarization.

4. The independent validation ultimately includes only 70 antibacterial pairs, which may be insufficient to robustly establish model generalizability across experimental platforms and scoring methodologies. A suitable justification is required for this.

5. Although the manuscript discusses prior models, direct benchmarking against contemporary approaches using the same curated dataset and identical pair-held-out validation schemes is lacking.

6. The rationale for selecting LightGBM as the primary model should be explained in greater detail, particularly relative to graph neural networks and deep learning approaches.

7. It is unclear whether class imbalance handling techniques were considered during model training, particularly for external validation where synergistic interactions constitute only 19% of samples.

8. Authors can also improve the overall introduction and discussion by incorporating recent literaure. For the same i have given few: PMID: 34365920, PMID: 34916893.

9. More information regarding the distribution of antibacterial classes in training and validation datasets would help assess potential chemical-space biases.

10. Exact p-values should be reported whenever statistical comparisons are performed.

Reviewer #2: The novelty of the proposed HALO framework appears to stem primarily from the evaluation strategy rather than the predictive model itself. The authors should more clearly distinguish the methodological novelty from existing Chemical Checker-based approaches and provide a direct comparison with state-of-the-art models under the same stringent validation framework.

The manuscript reports performance using a single curated dataset containing only 97 antibacterial compounds and 10 strains. The authors should discuss the limitations imposed by the relatively small chemical and biological diversity of the dataset and provide confidence intervals or statistical significance testing for the reported ROC-AUC values to better assess model robustness.

The manuscript excludes all interactions with |ε| ≤ 0.1 before classification, thereby removing a substantial proportion of biologically relevant near-additive interactions. The authors should quantify how many samples were excluded and evaluate whether the model remains effective under a more realistic multi-class or continuous prediction setting rather than only classifying extreme synergy and antagonism.

The authors claim that global feature selection in M3 introduces information leakage, but the extent of this leakage is not rigorously quantified. A formal comparison showing the number of selected features, overlap across folds, and statistical differences between M3 and HALO would strengthen the argument regarding the importance of fold-internal feature selection.

The manuscript uses gain-based LightGBM feature importance as the primary interpretability method. The authors should consider complementing this analysis with SHAP values or permutation-based importance because gain importance can be biased toward features with higher variability and may not accurately reflect biological relevance. Refer to this article: PMID-39657879 to improve the methodology, result, and discussion sections which will be more beneficial for the readership of the article and journal.

The authors conclude that strain embeddings contribute little predictive information. However, this conclusion may be confounded by sparse strain coverage and limited overlap of drug pairs across strains. Additional analyses examining strain-specific performance or incorporating genomic and phenotypic strain descriptors would be necessary before dismissing the value of strain-level information.

The manuscript reports successful external validation on the INDIGO dataset, yet only 70 antibacterial pairs remained after filtering. The authors should discuss the limited size of this validation cohort and evaluate whether any compounds or drug classes overlap with the training data, as such overlap could partially inflate external performance estimates.

The manuscript presents several high-confidence novel synergistic predictions and extensive mechanistic interpretations. However, no experimental validation is provided. The authors should moderate their conclusions and clearly state that these combinations remain computational hypotheses until verified through in vitro or in vivo testing.

Reviewer #3: The manuscript presents HALO, a machine learning framework that predicts antibacterial drug synergy using multi-level Chemical Checker bioactivity signatures. This study tackles a significant challenge in antimicrobial drug discovery by focusing on model evaluation without leakage. It also shows how bioactivity-based representations can help predict new antibacterial drug combinations. The manuscript is generally well organized, the methodology is clearly described, and its focus on thorough evaluation is a strong point. However, some issues should be addressed to enhance the scientific rigor and practical impact of the study. My main comments are listed below.

1. The dataset used for model development is relatively limited in size and diversity (97 antibacterial compounds and 3,160 drug–pair–strain interactions). Although the authors acknowledge this limitation, a more detailed discussion on how the dataset size may affect model generalizability and robustness is warranted. Additional validation on larger or more diverse datasets would strengthen the conclusions.

2. The manuscript primarily compares different evaluation schemes rather than benchmarking HALO against recent state-of-the-art antibacterial synergy prediction models. A direct comparison using the same dataset and evaluation protocol would provide a clearer assessment of its relative performance.

3. While the study emphasizes leakage-free evaluation, it remains unclear whether structurally or mechanistically similar drug pairs could still appear across training and test folds. The authors should discuss the potential influence of chemically related compounds on model performance and whether scaffold- or mechanism-based splitting strategies were considered.

4. The external validation is performed on only 70 drug pairs from the INDIGO dataset. Although encouraging, this relatively small external benchmark limits the strength of the generalization claims. Validation on additional independent datasets, bacterial species, or experimental platforms would strengthen the MS.

5. The model predicts binary synergy versus antagonism after excluding additive interactions (|ε| ≤ 0.1). Since most experimentally observed drug combinations are additive, excluding this large category may reduce the model's practical applicability. The authors should discuss this limitation and consider evaluating multi-class or continuous interaction prediction in greater detail.

6. Experimental validation of at least a subset of the newly predicted synergistic combinations would substantially increase the biological significance and translational impact of the work.

7. The manuscript concludes that strain embeddings contribute minimally to prediction; however, this may largely reflect the limited strain coverage rather than the lack of biological relevance. The authors should discuss whether richer strain-specific features, such as genomic, transcriptomic, or metabolic profiles, could improve predictive performance.

8. Although LightGBM was selected as the final model, the manuscript provides only limited justification for this choice. A more comprehensive comparison with recent deep learning approaches (e.g., graph neural networks or transformer-based architectures) under identical validation settings would strengthen the methodological rationale.

9. While the proposed framework is computationally efficient compared to deep learning methods, the manuscript does not provide information regarding computational cost, runtime, or scalability.

**********

**Have the authors made all data and (if applicable) computational code underlying the findings in their manuscript fully available?**

The PLOS Data policy requires authors to make all data and code underlying the findings described in their manuscript fully available without restriction, with rare exception (please refer to the Data Availability Statement in the manuscript PDF file). The data and code should be provided as part of the manuscript or its supporting information, or deposited to a public repository. For example, in addition to summary statistics, the data points behind means, medians and variance measures should be available. If there are restrictions on publicly sharing data or code —e.g. participant privacy or use of data from a third party—those must be specified.

Reviewer #1: Yes

Reviewer #2: Yes

Reviewer #3: Yes

**********

PLOS authors have the option to publish the peer review history of their article (what does this mean?). If published, this will include your full peer review and any attached files.

If you choose “no”, your identity will remain anonymous but your review may still be made public.

**Do you want your identity to be public for this peer review?**  For information about this choice, including consent withdrawal, please see our Privacy Policy.

Reviewer #1: No

Reviewer #2: No

Reviewer #3: No

[NOTE: If reviewer comments were submitted as an attachment file, they will be attached to this email and accessible via the submission site. Please log into your account, locate the manuscript record, and check for the action link "View Attachments". If this link does not appear, there are no attachment files.]

**Figure resubmission:**

**Reproducibility:**

?>

# Author comment: Author response to Decision Letter 1

**DOI:** 10.1371/journal.pcbi.1014849.r002

4 Sep 2026

Attachment Submitted filename: Response to Reviewers.docx

# Peer review: Decision Letter 1

**DOI:** 10.1371/journal.pcbi.1014849.r003

**Authors:** James Faeder, Rahul Singh

22 Sep 2026

Dear Dr. Mehrmohamadi,

We are pleased to inform you that your manuscript 'Bioactivity-Driven Prediction of Antibacterial Synergy Using Machine Learning Models' has been provisionally accepted for publication in PLOS Computational Biology.

Before your manuscript can be formally accepted you will need to complete some formatting changes, which you will receive in a follow up email. A member of our team will be in touch with a set of requests.

Please note that your manuscript will not be scheduled for publication until you have made the required changes, so a swift response is appreciated.

IMPORTANT: The editorial review process is now complete. PLOS will only permit corrections to spelling, formatting or significant scientific errors from this point onwards. Requests for major changes, or any which affect the scientific understanding of your work, will cause delays to the publication date of your manuscript.

Should you, your institution's press office or the journal office choose to press release your paper, you will automatically be opted out of early publication. We ask that you notify us now if you or your institution is planning to press release the article. All press must be co-ordinated with PLOS.

Thank you again for supporting Open Access publishing; we are looking forward to publishing your work in PLOS Computational Biology.

Best regards,

Rahul Singh, Ph.D.

Academic Editor

PLOS Computational Biology

James Faeder

Section Editor

PLOS Computational Biology

***********************************************************

Reviewer's Responses to Questions

**Comments to the Authors:**

**Please note here if the review is uploaded as an attachment.**

Reviewer #1: Authors have addressed all the comments.

Reviewer #2: The reviewers have responded well to the queries and questions of the reviewers. The manuscript is now worthy of publication.

Reviewer #3: The authors have satisfactorily addressed the comments and incorporated the necessary revisions into the manuscript. The revised manuscript is now suitable for acceptance and publication in its present form.

**********

**Have the authors made all data and (if applicable) computational code underlying the findings in their manuscript fully available?**

The PLOS Data policy requires authors to make all data and code underlying the findings described in their manuscript fully available without restriction, with rare exception (please refer to the Data Availability Statement in the manuscript PDF file). The data and code should be provided as part of the manuscript or its supporting information, or deposited to a public repository. For example, in addition to summary statistics, the data points behind means, medians and variance measures should be available. If there are restrictions on publicly sharing data or code —e.g. participant privacy or use of data from a third party—those must be specified.

Reviewer #1: **No:**

Reviewer #2: None

Reviewer #3: Yes

**********

PLOS authors have the option to publish the peer review history of their article (what does this mean?). If published, this will include your full peer review and any attached files.

If you choose “no”, your identity will remain anonymous but your review may still be made public.

**Do you want your identity to be public for this peer review?**  For information about this choice, including consent withdrawal, please see our Privacy Policy.

Reviewer #1: No

Reviewer #2: No

Reviewer #3: No

# Editor report: Acceptance letter

**DOI:** 10.1371/journal.pcbi.1014849.r004

**Authors:** James Faeder, Rahul Singh

PCOMPBIOL-D-26-01374R1

Bioactivity-Driven Prediction of Antibacterial Synergy Using Machine Learning Models

Dear Dr Mehrmohamadi,

I am pleased to inform you that your manuscript has been formally accepted for publication in PLOS Computational Biology. Your manuscript is now with our production department and you will be notified of the publication date in due course.

The corresponding author will soon be receiving a typeset proof for review, to ensure errors have not been introduced during production. Please review the PDF proof of your manuscript carefully, as this is the last chance to correct any errors. Please note that major changes, or those which affect the scientific understanding of the work, will likely cause delays to the publication date of your manuscript.

Soon after your final files are uploaded, unless you have opted out, the early version of your manuscript will be published online. The date of the early version will be your article's publication date. The final article will be published to the same URL, and all versions of the paper will be accessible to readers.

For Research, Software, and Methods articles, you will receive an invoice from PLOS for your publication fee after your manuscript has reached the completed accept phase. If you receive an email requesting payment before acceptance or for any other service, this may be a phishing scheme. Learn how to identify phishing emails and protect your accounts at https://explore.plos.org/phishing.

Thank you again for supporting PLOS Computational Biology and open-access publishing. We are looking forward to publishing your work!

With kind regards,

Sharmila Kamatchi

PLOS Computational Biology | Carlyle House, Carlyle Road, Cambridge CB4 3DN | United Kingdom ploscompbiol@plos.org | Phone +44 (0) 1223-442824 | ploscompbiol.org | @PLOSCompBiol
